# Comparative Analysis of Data Processing Tools for Mass Spectrometry Proteomics in Cardiovascular Research

**DOI:** 10.1021/acs.jproteome.6c00019

**Published:** 2026-07-31

**Authors:** Thakorn Pruktanakul, Steven Lynham, Honglin Chen, Xiaoke Yin, Manuel Mayr, Konstantinos Theofilatos

**Affiliations:** † King’s British Heart Foundation Centre, 170742 King’s College London, London SE5 9NU, U.K.; ‡ Proteomics Core Facility, King’s College London, London SE5 9NU, U.K.; § National Heart and Lung Institute, 4615 Imperial College London, London W12 0BZ, U.K.; ∥ Division of Internal Medicine, Faculty of Medicine, Prince of Songkla University, Songkhla 90110, Thailand

**Keywords:** cardiovascular, mass spectrometry, proteomics, data processing, data-dependent acquisition, data-independent acquisition

## Abstract

Mass spectrometry-based proteomics requires advanced data processing tools, yet most comparative studies have relied on noncardiovascular data sets or standard protein mixtures, limiting their relevance to cardiovascular research. We systematically compared three label-free data-dependent acquisition (DDA) tools (FragPipe, MaxQuant, and Proteome Discoverer) and five data-independent acquisition (DIA) tools (DIA-NN, DIA-Umpire, MSFragger-DIA, MaxDIA, and Spectronaut) using real-world cardiovascular tissue and blood-derived proteomics data sets. FragPipe and Spectronaut generally achieved the greatest quantitative proteome coverage among the evaluated DDA and DIA tools, respectively, particularly in cardiovascular tissue data sets, although tool rankings varied across sample types and proteome subsets. Despite differences in identification and quantification performance, peptide physicochemical characteristics were broadly similar across tools. Integration of complementary differential expression outputs increased differentially expressed protein detection in several data sets while maintaining agreement with the main results. These findings provide practical guidance for selecting data processing tools in cardiovascular proteomics and support the use of integrative approaches to maximize biological information from proteomics data sets.

## Graphical abstract

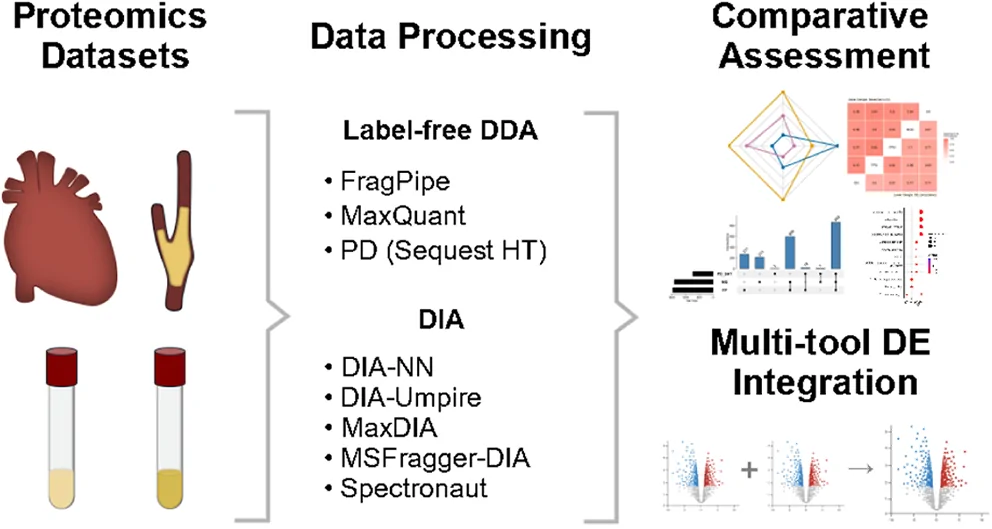

## Introduction

Mass spectrometry (MS)-based proteomics is a powerful approach for large-scale protein identification and quantification in biological samples.^1^ Its discovery approach aims to detect as many proteins as possible, providing a critical foundation for understanding biological mechanisms and identifying potential biomarkers.^2^ This is particularly relevant for diseases such as cardiovascular disease,^3^ which remains a leading cause of morbidity and mortality worldwide.

In addition to advances in MS technologies, bioinformatics approaches play essential roles in extracting meaningful information from proteomics data through optimized processing and analysis workflows. A recent study demonstrated that optimizing bioinformatics workflows can improve protein identification and maximize differential expression results,^4^ highlighting the value of enhancing bioinformatics in proteomics.

Raw MS data processing is a critical stage that converts complex MS signals into protein-level information, substantially influencing downstream analyses.^4^ This step is inherently complex and variable, largely shaped by acquisition strategies such as data-dependent acquisition (DDA) and data-independent acquisition (DIA).^5^ Numerous software platforms have been developed for liquid chromatography-tandem MS (LC-MS/MS) data processing. Some, such as MaxQuant,^6,7^ FragPipe,^8–12^ and Proteome Discoverer (PD, Thermo Fisher Scientific), can be applied to both DDA and DIA workflows, whereas the Trans-Proteomic Pipeline (TPP)^13^ is primarily for DDA. For DIA-specific analyses, tools such as DIA-NN,^14^ EncyclopeDIA,^15^ and Spectronaut (Biognosys) have been developed.

Several benchmarking studies have guided tool selection for commonly used MS data, showing that each platform has distinct strengths and weaknesses that depend on factors such as sample type, spectral library, and software version.^16–22^ Overall, PD has often been recommended for label-free DDA^17,19^ and DIA-NN for DIA.^20,21^ However, most evaluations have focused on multispecies standard protein mixtures or noncardiovascular tissues, limiting their relevance to cardiovascular proteomics. Cardiovascular tissue proteomes are highly complex, with heterogeneous cellular composition, unique cell phenotypes absent in other tissues,^23,24^ and a versatile extracellular matrisome. Consequently, studies based on cancer tissues or standard protein mixtures cannot reliably determine the best-performing tools for cardiovascular samples. Similarly, blood-based benchmarking requires separate consideration due to major differences in protein dynamic ranges compared with tissue.

Moreover, most proteomics studies rely on a single processing tool,^25–34^ which may miss disease-relevant proteins due to tool-specific limitations. The potential benefit of integrating differential expression outputs across tools has rarely been assessed. One study in multispecies standard protein mixtures reported promising results,^4^ but this approach has not yet been evaluated in real-world cardiovascular proteomics data sets.

In this study, we systematically compared widely used processing tools for discovery LC-MS/MS proteomics using data sets covering two common acquisition strategies and including both cardiovascular tissues and blood-derived samples. We evaluated MaxQuant, FragPipe, TPP, and PD for label-free DDA data, and MaxDIA, MSFragger-DIA, DIA-Umpire, DIA-NN, and Spectronaut for DIA data. Additionally, we developed and assessed a workflow for integrating differential expression outputs across tools. Our study provides practical guidance for tool selection and introduces a strategy to enhance the detection of dysregulated proteins in cardiovascular proteomics.

## Experimental Procedures

### Experimental Design for LC-MS/MS Processing Tool Comparison

Human LC-MS/MS data sets were analyzed using processing tools compatible with each acquisition strategy, employing the latest software versions available at the start of the study. Comparative assessments spanned multiple analytical dimensions, including proteome identification, quantification performance, and downstream functional analyses. A schematic overview of the workflow is provided in Figure 1.

**Figure 1:**
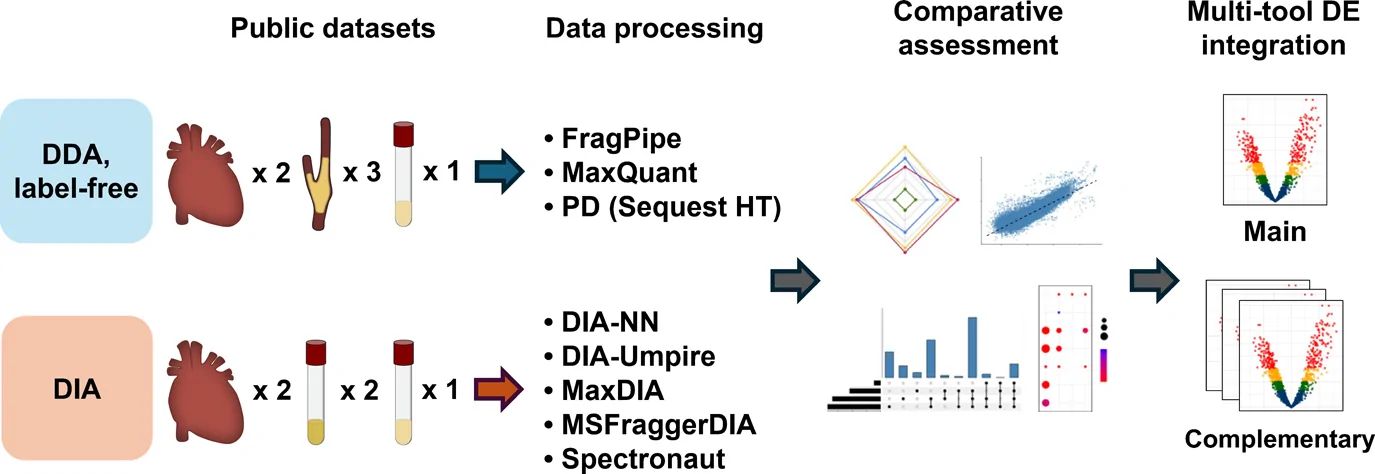
Study workflow. The study included six label-free DDA and five DIA cardiovascular proteomics data sets derived from human samples. Comparative analyses assessed tool performance and peptide physicochemical properties, while integration analyses evaluated tool agreement and integrated differential expression outputs.

Raw human proteomics data were obtained from ProteomeXchange, comprising six label-free DDA data sets and five DIA data sets from cardiac tissue, vascular tissue, serum, and plasma. All data sets originated from cardiovascular studies, except for three blood-based DIA data sets derived from COVID-19 and multisystem inflammatory syndrome (MIS-C) cohorts. This substitution was necessary because no publicly available blood-based DIA proteomics data sets include sample-level cardiovascular annotations, and the selected conditions represent systemic inflammatory states similar to those observed in active cardiovascular disease. To avoid potential confounding effects of postmortem degradation and inflammation,^21^ only data from fresh-frozen cardiovascular samples were included.

For label-free DDA, PXD053721 included 64 raw files from left ventricular biopsies of patients with advanced heart failure (HF) (n = 48) and nonfailing controls (n = 16).^34^ PXD060431 included 30 raw files from right ventricular septal biopsies of patients with HF with preserved ejection fraction (n = 10), HF with reduced ejection fraction (n = 10), and nonfailing controls (n = 10).^35^ PXD035563 included 36 raw files from carotid endarterectomy (CEA) specimens of patients with carotid artery stenosis (n = 12), representing 24 atherosclerotic regions and 12 paired nondiseased regions.^31^ PXD005130 comprised two sets of 384 raw files from two replicates, each with 16 fractions of NaCl-extracted (soluble matrisome) and GuHCl-extracted (core matrisome) CEA specimens from symptomatic (n = 6) and asymptomatic (n = 6) carotid artery stenosis.^25^ PXD008278 comprised 84 raw files from serum samples of patients with ultrasound-detected carotid plaque (n = 42) and matched plaque-free controls (n = 42).^26^ A summary of these data sets is provided in Table 1.

**Table 1:** Summary of Label-Free DDA Datasets^a^

Sample type	MS instrument	No. of raw files	Conditions (sample size)	Accession (citation)
Cardiac tissue	Q Exactive HF-X Orbitrap	64	Advanced HF (48)	PXD053721 (Beyhoff et al.^34^)
			Nonfailing controls (16)	
Cardiac tissue	Orbitrap Exploris 480	30	HFpEF (10)	PXD060431 (Jani et al.^35^)
			HFrEF (10)	
			Nonfailing controls (10)	
CEA tissue	Orbitrap Fusion Lumos	36	Plaque regions (12)	PXD035563 (Blaser et al.^31^)
			Control regions (12)	
CEA tissue, NaCl-extracted	LTQ Orbitrap XL	384	Symptomatic CAS (6)	PXD005130 (Langley et al.^25^)
			Asymptomatic CAS (6)	
CEA tissue, GuHCl-extracted	LTQ Orbitrap XL	384	Symptomatic CAS (6)	PXD005130 (Langley et al.^25^)
			Asymptomatic CAS (6)	
Serum	LTQ Orbitrap Velos Pro	84	US-detected carotid plaques (42)	PXD008278 (Bhosale et al.^26^)
			Matched controls (42)	

a Abbreviations: CAS, carotid artery stenosis; CEA, carotid endarterectomy; HF, heart failure; HFpEF, HF with preserved ejection fraction; HFrEF, HF with reduced ejection fraction; US, ultrasound.

For DIA, PXD018678 consisted of 30 raw files from left ventricular samples of patients with ischemic cardiomyopathy (n = 15) and matched donor controls (n = 15).^27^ PXD050610 comprised 25 raw files from left ventricular samples of older donors (≥50 years; n = 13) and younger donors (≤25 years; n = 12).^36^ PXD025462 included 49 raw files from serum samples of children with MIS-C (n = 25) and healthy controls (n = 24).^30^ PXD047901 comprised 36 raw files, each with three fractions, from plasma samples of severe or critical coronavirus disease 2019 (COVID-19) patients (n = 8) and non-COVID-19 respiratory failure controls (n = 4).^33^ PXD024089 consisted of 59 raw files from plasma samples spiked with PQ500 reference peptides of COVID-19 intensive care unit (ICU) patients (n = 48) and sepsis ICU patients (n = 11).^29^ A summary of these data sets is provided in Table 2.

**Table 2:** Summary of DIA Datasets^a^

Sample type	MS instrument	No. of raw files	Conditions (sample size)	Accession (citation)
Cardiac tissue	Q Exactive HF-X Orbitrap	30	ICM (15)	PXD018678 (Li et al.^27^)
			Healthy controls (15)	
Cardiac tissue	Orbitrap Fusion Lumos	25	Older donors (13)	PXD050610 (Malecki et al.^36^)
			Younger donors (12)	
Serum	Orbitrap Exploris 480	49	MIS-C (25)	PXD025462 (Porritt et al.^30^)
			Healthy controls (24)	
Plasma	Orbitrap Exploris 240	36	Severe COVID-19 (8)	PXD047901 (Ward et al.^33^)
			RF controls (4)	
Plasma, PQ500-spiked	Orbitrap Fusion Lumos	59	COVID-19 ICU (48)	PXD024089 (Gutmann et al.^29^)
			Sepsis ICU (11)	

a Abbreviations: COVID-19, coronavirus disease 2019; ICM, ischemic cardiomyopathy; ICU, intensive care unit; MIS-C, multisystem inflammatory syndrome in children; RF, respiratory failure.

All data sets analyzed in this study were obtained from previously published studies and publicly available repositories. No new human participants or animals were recruited, and no new biological samples were collected as part of this study. Ethical approval and informed consent, where applicable, were obtained by the investigators of the original studies, as described in the corresponding publications.

The tools evaluated in this study were chosen based on their widespread use and compatibility with the acquisition strategies analyzed. For DDA data, we included MaxQuant (version 2.7.5.0) and the Sequest HT workflow implemented in PD (PD_SHT; version 3.2.0) because they were widely used in previous benchmarking studies.^17,19^ FragPipe (version 24.0) and TPP (version 7.3) were also selected due to their active development and broad community adoption.^11–13^ However, TPP was subsequently excluded because it could not reliably complete analyses of the large-scale data sets included in this study, owing to repeated server timeouts.

For DIA data, we focused on library-free tools, as experimental spectral libraries were not available for all data sets. Although experimental libraries generally provide more robust results,^21^ another study shows that modern DIA tools with in silico libraries achieve comparable or superior coverage and accuracy with greater speed and cost-efficiency.^20^ The DIA tools included MaxDIA in MaxQuant (version 2.7.5.0), MSFragger-DIA and DIA-Umpire in FragPipe (version 24.0), DIA-NN (version 2.5.1), and Spectronaut (version 20.3), selected for their widespread use and frequent citation in the literature.^37^

Compatible workflows and bundled tools are listed in Supplemental Table S1. All analyses were conducted on a workstation equipped with an Intel Xeon Gold 5118 CPU (12 cores, 24 threads) and 128 GB RAM running Windows 10 Enterprise. Due to licensing requirements, PD and Spectronaut were executed on separate dedicated workstations. To ensure transparency and reproducibility, data analysis workflows and methods were reported in accordance with the recommendations of Doncheva et al.^38^ where applicable.

### Protein and Peptide Sequence Databases

Human protein sequences were retrieved from UniProt/SwissProt human protein database (UP000005640_9606; release 2024_06), comprising 20,328 entries, and used for all analyses. iRT peptide sequences compatible with the PQ500 SIS mix (Biognosys AG, Ki-3019-96), as in the original study, were downloaded from the manufacturer’s website (accessed 24 April 2025) and included in all DIA analyses.

For FragPipe, decoy and common contaminant sequences were appended to the human protein database using the built-in tool prior to DDA analysis. In DIA workflows, the human and iRT peptide sequences were combined, with decoy and contaminant sequences appended via the Philosopher executable through the Windows command line.

### Data Format Conversion

All raw files (.raw) were directly imported into MaxQuant, PD, and Spectronaut. For the remaining tools, raw files were converted to mzML format using MSConvert from ProteoWizard (version 3.0.24310), applying vendor-specific peak picking for both DDA and DIA data. This approach was used for all DIA data sets because their isolation windows overlapped by less than 50% of their width.

### Spectral Library Generation for DIA

Spectral libraries were generated using tool-specific, DDA-independent approaches. For MaxDIA, libraries were predicted in silico from the human protein database using the predicted mode, while DIA-NN employed its library-free mode. In FragPipe, libraries were derived from the DIA data using the DIA_SpecLib_Quant workflow with MSFragger-DIA and the DIA_DIA-Umpire_SpecLib_Quant workflow with DIA-Umpire. Spectronaut generated libraries directly from DIA data using the directDIA+ (Deep) workflow.

### Tool Parameter Settings

Key parameters related to database searching, protein digestion, and fixed and variable modifications were adopted from the original analyses of each data set, as summarized in Supplemental Tables S2 and S3. A false discovery rate (FDR) of 1% was applied at all available levels. For all label-free DDA analyses, the match-between-runs feature was enabled in MaxQuant and FragPipe, while the Feature Mapper module was enabled in PD_SHT. In the PQ500-spiked DIA analysis, Spectronaut used its PQ500 plug-in, whereas all other tools relied on SILAC settings to distinguish heavy-arginine and heavy-lysine peptides from light peptides. Unless otherwise specified, all other parameters were kept at their default values.

### Evaluation Metrics

Identification performance was evaluated based on the number of stripped peptide sequences and representative proteins. Representative proteins were defined as the software-designated leading or master proteins representing protein groups and were used to standardize protein identifications across software tools.

Quantification performance was assessed using two metrics: the percentage of missing values among representative proteins within each data set, and the number of quantifiable proteins, defined as representative proteins with measurable intensities in ≥70% of samples. Tool-specific proteins were defined as quantifiable proteins uniquely identified by a single tool within each data set.

For the PQ500-spiked plasma data set, quantification performance was evaluated using the heavy reference peptides included in the PQ500 standard. Known concentrations were available for the heavy reference peptides, which were spiked into all samples at identical amounts, but not for the corresponding endogenous peptides. Therefore, absolute quantification accuracy could not be assessed. Instead, peptide recovery and quantitative reproducibility were evaluated. Peptide recovery was calculated as the percentage of PQ500 heavy reference peptides detected after tool-specific confidence filtering, whereas quantitative reproducibility was assessed using the median coefficient of variation (CV) of peptide abundances across samples.

Computational demand was assessed using total runtime and output file size. Proteome Discoverer and Spectronaut were excluded from this analysis because they were executed on different workstations, precluding a fair comparison. User experience was qualitatively evaluated based on the clarity of user documentation, interface usability, and software stability.

### Peptide Physicochemical Properties

Peptide physicochemical properties were evaluated to assess whether differences in peptide characteristics contributed to tool-specific identification and quantification performance. Peptide length, amino acid composition, and precursor charge-state distributions were calculated from peptide identification results. Peptide hydrophobicity (Kyte-Doolittle scale) and isoelectric point (pI) were calculated from identified peptide sequences using the Peptides^39^ package in R.

### Protein Molecular Weight Distribution

Protein molecular weight (MW) distributions were evaluated to determine whether tools differed in the sizes of proteins identified and quantified across data sets. Protein MW values were obtained through UniProt^40^ ID mapping for representative and quantifiable proteins identified by each tool.

### Tool-Specific Protein Identification

Shared and tool-specific protein sets were generated by intersecting quantifiable protein lists across tools within each data set. Proteins quantified by more than one tool were classified as shared proteins, whereas proteins quantified by only one tool were classified as tool-specific proteins. Identification confidence was assessed by comparing unique peptide counts and protein-level confidence metrics reported by each tool (e.g., probabilities, scores, or q-values) between shared and tool-specific proteins within each data set. Functional enrichment analyses were subsequently performed on tool-specific proteins to evaluate their potential biological characteristics.

### Multitool Integration of Differential Expression Outputs

To assess the potential benefit of incorporating tool-specific proteins into downstream analyses, differential expression outputs from multiple tools were integrated using an agreement-based strategy. For each data set, the tool yielding the highest number of quantifiable proteins was designated as the main result to maximize quantitative proteome coverage. The only exception was the PQ500-spiked plasma data set, where Spectronaut was designated as the main result due to its dedicated support for PQ500 data processing.

Outputs from other tools were incorporated only if they met the predefined agreement criteria of at least moderate Spearman abundance correlation (ρ > 0.4) and ≥70% directional concordance with the main results. These thresholds were selected to balance the inclusion of complementary tool outputs with consistency in protein abundance profiles and differential expression directions. Tool-specific proteins were identified for each tool and data set and subsequently combined with the main results.

The robustness of these criteria was evaluated through sensitivity analyses by varying the Spearman correlation (0.3–1.0) and directional concordance (0.6–1.0) thresholds and examining their impact on the inclusion of complementary tool outputs. Percentage increases were calculated relative to the number of significant differentially expressed proteins (DEPs) identified by the main processing tool.

### Statistical Rationale

Differences in data set-level median peptide length, hydrophobicity, and pI among tools were assessed separately for DDA and DIA tools using Friedman tests, treating data sets as matched blocks to account for the paired study design. Statistical significance was defined as *p* < 0.05.

Protein MW distributions were first assessed for normality using the Shapiro–Wilk test. Accordingly, differences among tools were evaluated using Kruskal–Wallis tests, followed by Dunn’s multiple-comparison procedure with Bonferroni correction.^41^ Statistical significance was defined as Bonferroni-adjusted *p* < 0.05.

Normalized protein intensities (DDA and DIA) or normalized light-to-heavy peptide intensity ratios (PQ500-spiked DIA) were log2-transformed and subjected to missing value imputation using the KNN method in the impute^42^ package for downstream analyses. Spearman’s rank correlation coefficient (ρ) was calculated both globally and on a per-protein basis across matched samples.

Differential expression analysis (DEA) was performed using the empirical Bayes framework implemented in the limma^43^ package. Differential expression was defined as a Benjamini-Hochberg^44^-adjusted p-value <0.05 and an absolute log2 fold change (log2FC) > 0.3. Directional concordance was calculated as the proportion of shared proteins with concordant log2FC directions across software tools within each data set.

Functional enrichment analyses of tool-specific proteins were performed using DAVID^45^ with the DAVID Knowledgebase (release 2026_1), and terms with a Benjamini-Hochberg-adjusted p-value <0.05 were considered significantly enriched.

All statistical analyses were performed in R (version 4.5.2; R Core Team, 2025). Data visualization was performed using the following R packages: fmsb^46^ for radar charts; ggplot2 ^47^ for box plots, enrichment plots, correlation plots, and volcano plots; pheatmap^48^ for heatmaps; and ComplexHeatmap^49^ for UpSet plots.

## Results and Discussion

### Proteome Identification and Quantification Performance of Label-Free DDA Tools

Across the two cardiac proteome data sets (Figure 2A–B), FragPipe yielded the highest numbers of quantifiable proteins (3,894 and 1,748), exceeding MaxQuant, the second-ranked tool, by 4–29%. This advantage was driven by greater peptide and protein identification depth, which outweighed its slightly higher missingness. In contrast, although PD_SHT identified more peptides and proteins than MaxQuant, its substantially higher missingness (47–67%) resulted in the fewest quantifiable proteins.

**Figure 2:**
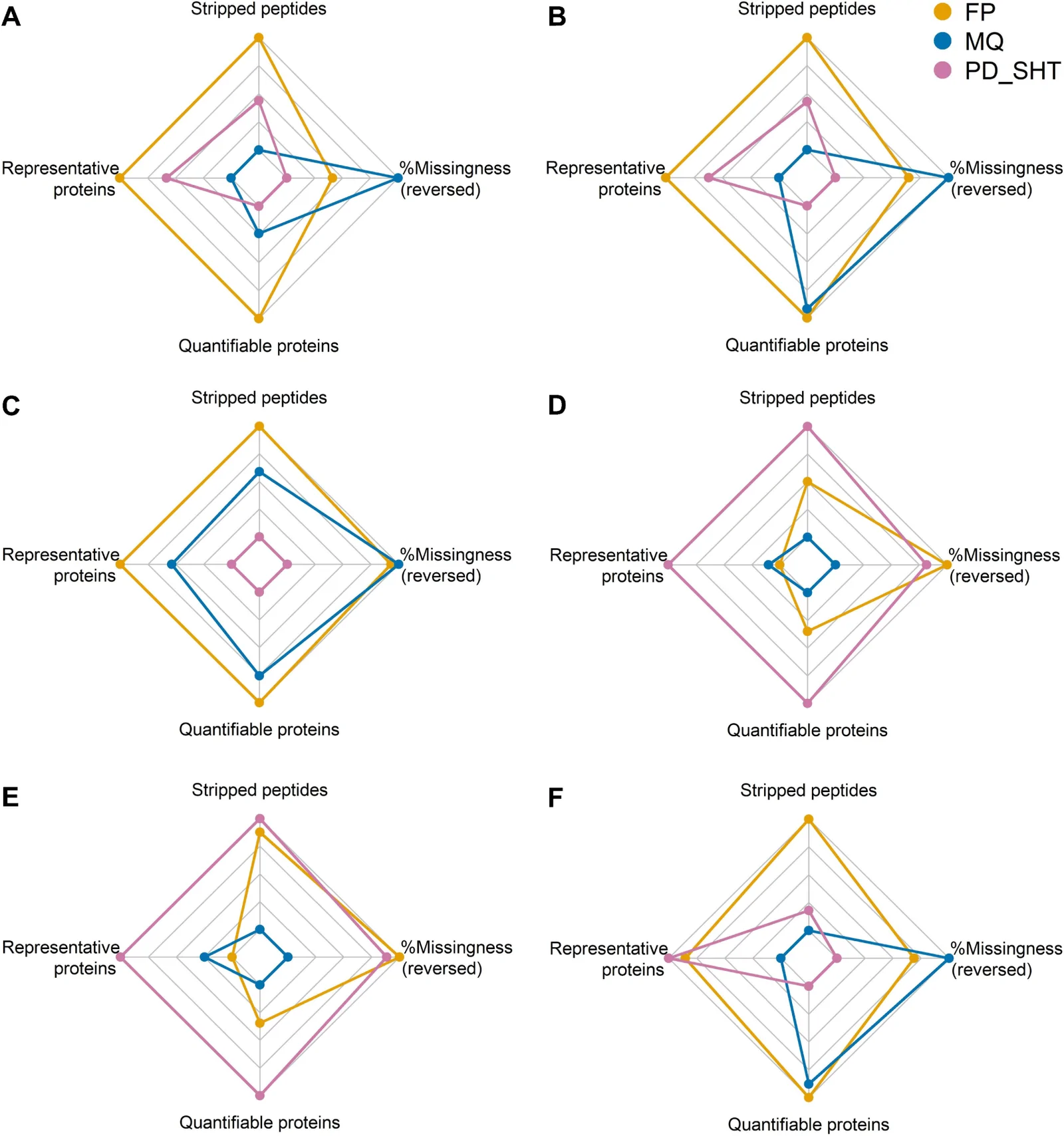
Comparative identification and quantification performance of label-free DDA tools. Radar charts show normalized values for stripped peptides, representative proteins, reversed missing-value percentage, and quantifiable proteins obtained using FragPipe (FP), MaxQuant (MQ), and Proteome Discoverer with Sequest HT (PD_SHT) across label-free DDA data sets from (A) cardiac proteome (PXD053721), (B) cardiac proteome (PXD060431), (C) vascular proteome (PXD035563), (D) core vascular matrisome (PXD005130), (E) soluble vascular matrisome (PXD005130), and (F) serum proteome (PXD008278). Normalized scores range from 0.0 to 1.0, with concentric polygons representing values of 0.0, 0.25, 0.50, 0.75, and 1.0 from the center outward.

A similar pattern was observed in the vascular proteome data set (Figure 2C), where FragPipe again generated the largest number of quantifiable proteins (1,453), exceeding MaxQuant by 27%. However, the trend differed in the vascular matrisome data sets (Figure 2D–E), where PD_SHT produced the highest numbers of quantifiable proteins (1,155 and 1,512), surpassing FragPipe by 58–83%. This superior performance was primarily attributable to greater peptide and protein identification depth while maintaining missingness levels comparable to those of FragPipe.

In the serum proteome data set (Figure 2F), FragPipe again produced the highest number of quantifiable proteins (198), although the difference from MaxQuant was negligible (0.1%). Despite achieving the greatest protein identification depth, PD_SHT exhibited the highest missingness (48%), resulting in the fewest quantifiable proteins, consistent with its performance in the cardiac and vascular tissue data sets.

Overall, FragPipe generally provided the highest quantitative proteome coverage across cardiovascular tissue and serum data sets, followed by MaxQuant and PD_SHT. An exception was observed in the vascular matrisome data sets, where PD_SHT achieved superior performance. Detailed performance metrics are summarized in Supplemental Table S4.

Across all label-free DDA data sets, FragPipe achieved the highest median numbers of stripped peptides (19,626), representative proteins (1,995), and quantifiable proteins (1,205), whereas PD_SHT exhibited the highest median missingness (47.5%) (Supplemental Table S5). Friedman tests identified a significant difference among tools only for stripped peptide counts, whereas representative protein counts, quantifiable protein counts, and missingness did not differ significantly. This may be attributed to the heterogeneous performance patterns observed across different proteome subsets. However, further subgroup analyses were not feasible because of the limited number of data sets available within each proteome subset.

### Proteome Identification and Quantification Performance of DIA Tools

Across the two cardiac proteome data sets (Figure 3A–B), Spectronaut yielded the highest numbers of quantifiable proteins (4,165 and 3,652), exceeding DIA-NN, the second-ranked tool, by 3–12%. This advantage was driven by both greater peptide and protein identification depth and lower missingness. Although DIA-NN exhibited the highest missingness (22% and 9%), its strong identification performance resulted in the second-highest quantitative proteome coverage, followed by MSFragger-DIA.

**Figure 3:**
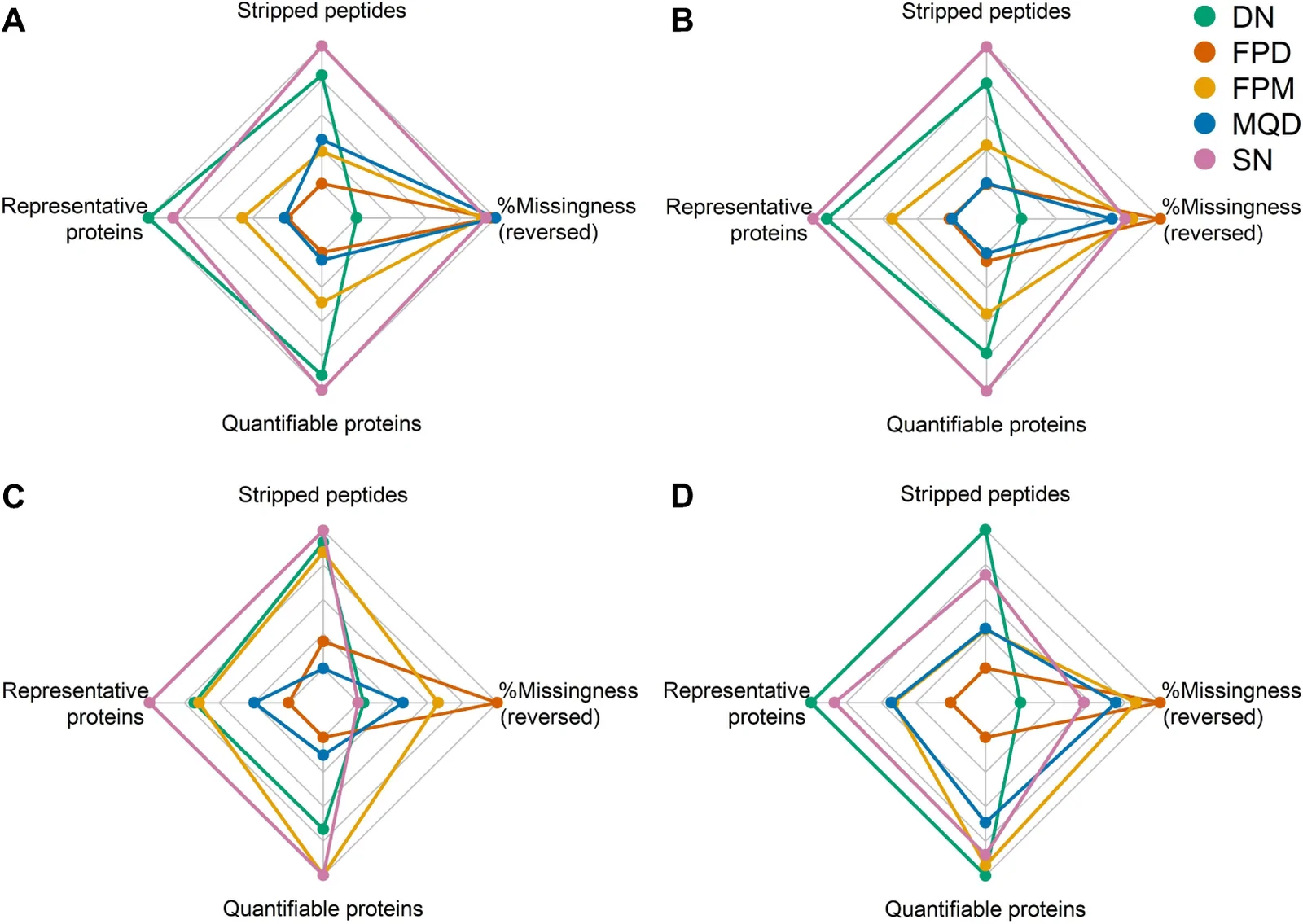
Comparative identification and quantification performance of DIA tools. Radar charts show normalized values for stripped peptides, representative proteins, reversed missing-value percentage, and quantifiable proteins obtained using DIA-NN (DN), DIA-Umpire (FPD), MaxDIA (MQD), MSFragger-DIA (FPM), and Spectronaut (SN) across DIA data sets from (A) cardiac proteome (PXD018678), (B) cardiac proteome (PXD050610), (C) serum proteome (PXD025462), and (D) plasma proteome (PXD047901). Normalized scores range from 0.0 to 1.0, with concentric polygons representing values of 0.0, 0.25, 0.50, 0.75, and 1.0 from the center outward.

In the serum proteome data set (Figure 3C), Spectronaut and MSFragger-DIA produced similarly high numbers of quantifiable proteins (383), exceeding DIA-NN by approximately 20%. In contrast, the plasma proteome data set (Figure 3D) showed a different pattern, with DIA-NN yielding the highest number of quantifiable proteins (728), surpassing MSFragger-DIA by 3%. Despite exhibiting the highest missingness (54%), DIA-NN achieved the greatest quantitative coverage because of its substantially higher peptide and protein identification depth.

Overall, Spectronaut, DIA-NN, and MSFragger-DIA consistently ranked among the top-performing DIA tools in terms of quantitative proteome coverage, although the leading tool varied by sample type. Spectronaut performed best for cardiac proteomes, Spectronaut and MSFragger-DIA performed similarly in serum, and DIA-NN achieved the greatest quantitative proteome coverage in plasma. Detailed performance metrics are summarized in Supplemental Table S4.

Consistent with these observations, summary statistics across all DIA data sets showed overall advantages for Spectronaut, which achieved the highest median stripped peptide count (22,038) and quantifiable protein count (2,169), whereas DIA-Umpire exhibited the lowest median missingness (4.0%) (Supplemental Table S5). DIA-NN also demonstrated comparable identification depth and quantitative proteome coverage across data sets.

### Physicochemical Characteristics of Peptides Identified across DDA and DIA Tools

Despite substantial differences in peptide and protein identifications, the physicochemical characteristics of identified peptides were broadly similar across processing tools (Supplemental Figures S1–S2; Supplemental Table S6). For label-free DDA analyses, median peptide lengths, hydrophobicity values, and pI values ranged from 13 to 14 amino acids, −0.32 to −0.27, and 6.06–6.33, respectively. For DIA analyses, the corresponding ranges were 12–13 amino acids, −0.33 to −0.28, and 5.86–6.27, respectively. Data set-level Friedman tests identified a statistically significant difference only for peptide hydrophobicity in DDA analyses; however, the magnitude of this difference was small relative to the overall range of observed values.

Collectively, these findings indicate that the evaluated tools identified peptides with highly similar physicochemical characteristics, which were predominantly moderately hydrophilic and near-neutral in pI, despite differences in peptide and protein identification performance.

### Protein Molecular Weight Distribution

Representative and quantifiable proteins exhibited broadly similar MW distributions across tools within each data set, with no consistent pattern favoring proteins of particular MW ranges (Supplemental Figures S3–S4 and Supplemental Tables S7–S8). These findings indicate that the evaluated tools showed no consistent bias toward proteins of specific molecular weight ranges.

### Influence of Proteome Composition on Tool Performance

Previous benchmarking studies have provided valuable guidance for selecting tools for label-free DDA and DIA workflows; however, most were performed using noncardiovascular data sets and did not evaluate FragPipe-based workflows, which have become widely adopted in modern proteomics.^17,19–21^ Consequently, the extent to which previously reported performance patterns generalize to cardiovascular proteomes remains uncertain. Although direct evidence for systematic differences in proteome characteristics across tissues is limited, large-scale single-cell atlases have demonstrated substantial variation in cellular composition among tissues,^50^ which is expected to influence underlying molecular profiles, including the proteome.

This study provides an updated assessment of data processing tool performance in cardiovascular proteomics across fresh-frozen cardiovascular tissues and blood-derived samples. The results suggest that tool performance tends to be more consistent among data sets representing similar proteome subsets, as observed for cardiac proteomes in both DDA and DIA analyses, where FragPipe and Spectronaut achieved the greatest quantitative proteome coverage, respectively (Figure 4). In contrast, distinct performance patterns were observed between different proteome subsets derived from the same biological source, as demonstrated by the vascular proteome and vascular matrisome DDA data sets as well as the serum and plasma DIA data sets. Together, these findings suggest that tool performance may be influenced by proteome composition.

### PQ500 Heavy Peptide Recovery and Quantitative Reproducibility

Assessment of quantitative performance using the PQ500 heavy reference peptides in one plasma data set (PXD024089) showed that, among the 804 reference peptides, DIA-Umpire and MSFragger-DIA achieved the highest recovery rates (94.2% and 93.3%), followed by DIA-NN (89.6%), Spectronaut (84.5%), and MaxDIA (84.2%) (Supplemental Table S9). However, quantitative reproducibility showed a different pattern, with Spectronaut and DIA-NN exhibiting the lowest median peptide-level CVs (13.3% and 15.0%, respectively), whereas DIA-Umpire and MSFragger-DIA showed moderately higher variability (22.4% and 22.3%, respectively). MaxDIA displayed substantially greater variability (52.9%).

Although PQ500 was originally developed for use within the Spectronaut ecosystem, the comparison was extended to other DIA tools to provide complementary evidence of tool-specific differences in peptide detection and quantitative reproducibility that could influence light-to-heavy ratio measurements and downstream DEA.

### Computational Demand and User Experience

Computational demand and user experience varied substantially across tools (Supplemental Figure 4; Supplemental Table S4). Among DDA tools, FragPipe exhibited the lowest computational demands, whereas PD_SHT provided the most favorable user experience. Among DIA tools, MSFragger-DIA and DIA-Umpire, both implemented within the FragPipe platform, exhibited the lowest computational demands, while MSFragger-DIA, DIA-Umpire, and Spectronaut provided the most user-friendly interfaces and workflow configurations. These observations highlight practical considerations that may influence tool selection alongside analytical performance.

**Figure 4:**
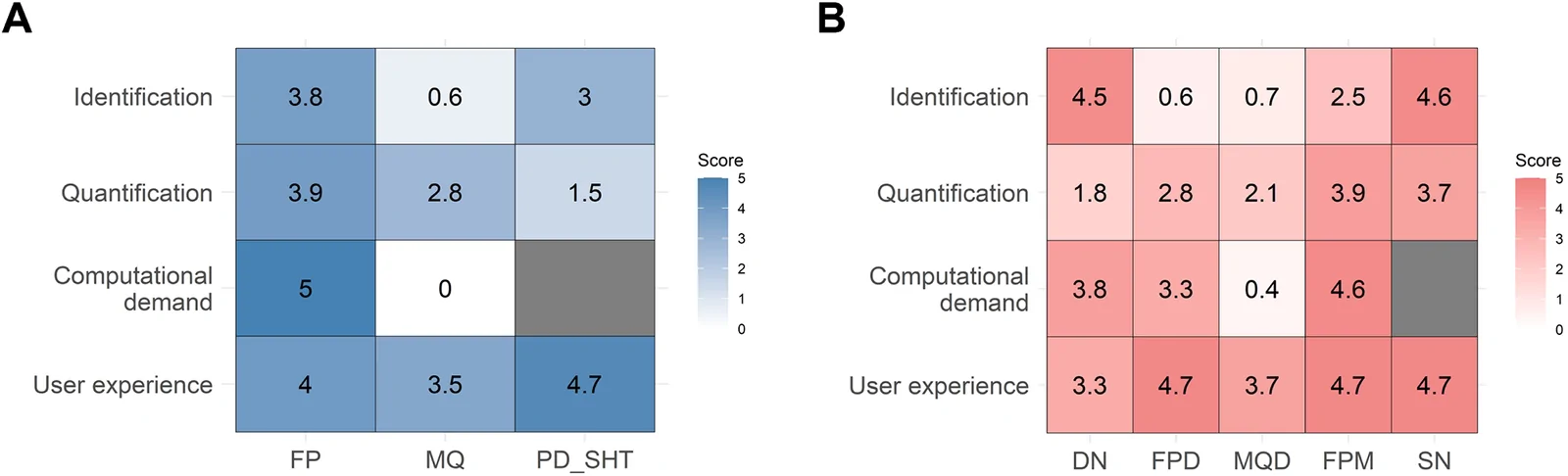
Comparative evaluation of performance, computational demand, and user experience across tools. Heatmaps display normalized scores (0–5) for identification (stripped peptides and representative proteins), quantification (% missing values and number of quantifiable proteins), computational demand (run time and output size), and user experience (clarity of user documentation, interface usability, and software stability). Results are shown for (A) label-free DDA tools: FragPipe (FP), MaxQuant (MQ), and Proteome Discoverer with Sequest HT (PD_SHT); and (B) DIA tools: MaxDIA (MQD), MSFragger-DIA (FPM), DIA-Umpire (FPD), DIA-NN (DN), and Spectronaut (SN). PD_SHT and SN were excluded from the computational demand analysis because they were executed on a different workstation.

### Tool-Specific Proteins, Identification Confidence, and Functional Characteristics

In the label-free DDA data sets, tool-specific proteins accounted for 15.9–46.3% of the total protein union, with the highest proportions observed in the vascular matrisome data sets (Figure 5; Supplemental Table S10). FragPipe consistently yielded the largest numbers of tool-specific proteins in the cardiac, vascular, and serum proteome data sets, whereas PD_SHT contributed the largest tool-specific protein sets in both vascular matrisome data sets.

**Figure 5:**
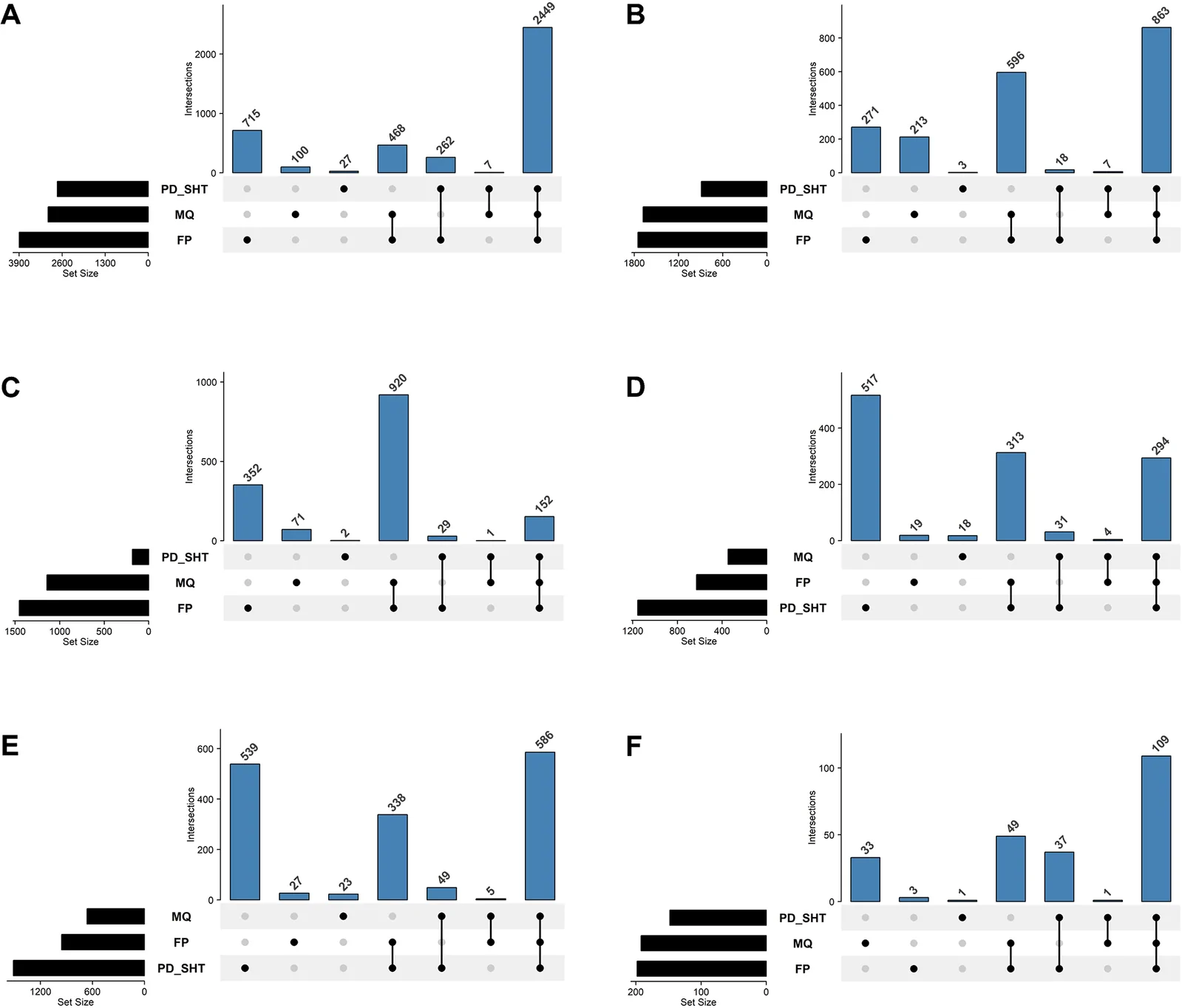
Tool-specific and shared protein identifications across label-free DDA tools. UpSet plots illustrate protein intersections obtained using FragPipe (FP), MaxQuant (MQ), and Proteome Discoverer with Sequest HT (PD_SHT) across label-free DDA data sets from (A) cardiac proteome (PXD053721), (B) cardiac proteome (PXD060431), (C) vascular proteome (PXD035563), (D) core vascular matrisome (PXD005130), (E) soluble vascular matrisome (PXD005130), and (F) serum proteome (PXD008278).

In the DIA data sets, tool-specific proteins accounted for 14.4–35.6% of the total protein union (Figure 6; Supplemental Table S10). Spectronaut generated the largest numbers of tool-specific proteins in most DIA data sets, particularly the cardiac tissue and serum proteomes, whereas DIA-NN and MSFragger-DIA also contributed substantial numbers of tool-specific proteins across data sets.

**Figure 6:**
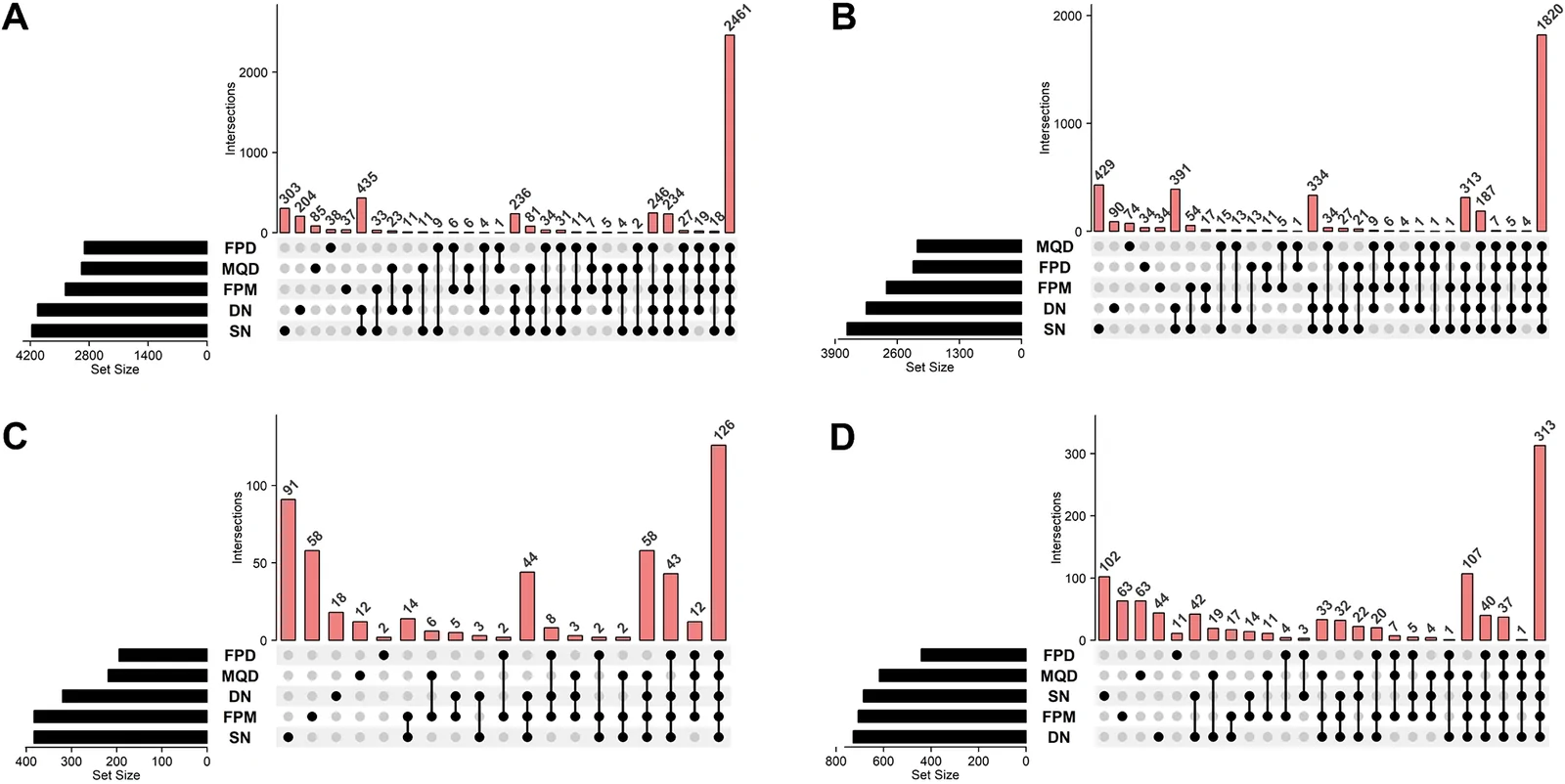
Tool-specific and shared protein identifications across DIA tools. UpSet plots illustrate protein intersections obtained using DIA-NN (DN), DIA-Umpire (FPD), MaxDIA (MQD), MSFragger-DIA (FPM), and Spectronaut (SN) across DIA data sets from (A) cardiac proteome (PXD018678), (B) cardiac proteome (PXD050610), (C) serum proteome (PXD025462), and (D) plasma proteome (PXD047901).

Although shared proteins constituted the largest intersection in all data sets, substantial fractions of the quantifiable proteome remained tool-specific across both acquisition strategies. These findings indicate that relying on a single processing tool, even one providing the highest quantitative coverage, may overlook proteins uniquely quantified by alternative tools.

To assess the confidence of tool-specific proteins, unique peptide counts and tool-specific confidence metrics were compared between shared and tool-specific proteins. Across both DDA and DIA data sets, tool-specific proteins generally exhibited lower peptide support and confidence metrics than shared proteins (Supplemental Tables S11–S12), suggesting weaker identification support overall. However, they remained supported by the confidence standards implemented within each tool, satisfying the corresponding confidence criteria and 1% FDR filtering thresholds.

Functional enrichment analysis of tool-specific proteins revealed distinct biological themes among tools within several data sets. In the cardiac DDA data set (Figure 7A), FragPipe-specific proteins were enriched for mitochondrial and protein transport functions, whereas MaxQuant-specific proteins highlighted extracellular vesicle and Rho GTPase signaling pathways. More pronounced differences were observed in the core vascular matrisome data set (Figure 7B), where FragPipe-specific proteins were associated with blood microparticle and immunoglobulin complex terms, MaxQuant-specific proteins with immune-related pathways, and PD-specific proteins with focal adhesion and RNA-binding functions.

**Figure 7:**
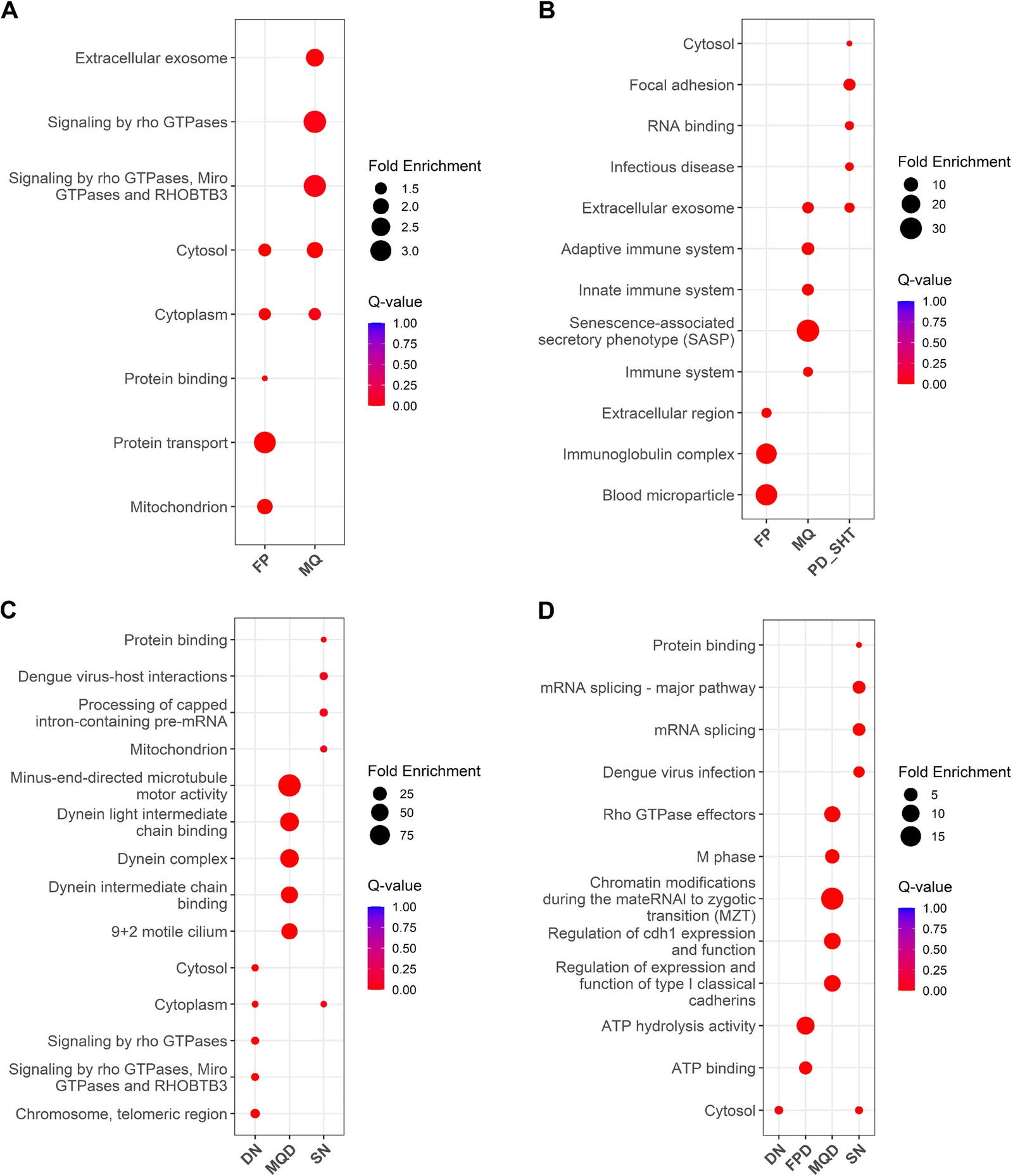
Functional enrichment analysis of tool-specific proteins identified by different processing tools. The five most significantly enriched functional terms associated with tool-specific proteins are shown for label-free DDA tools, including FragPipe (FP), MaxQuant (MQ), and Proteome Discoverer with Sequest HT (PD_SHT) in (A) cardiac proteome (PXD053721) an dB) core vascular matrisome (PXD005130), and for DIA tools, including DIA-NN (DN), DIA-Umpire (FPD), MaxDIA (MQD), and Spectronaut (SN) in (C) cardiac proteome (PXD018678) and (D) cardiac proteome (PXD050610).(

Similarly, in the DIA cardiac data sets, different tools highlighted distinct biological themes, including chromosome organization and Rho GTPase signaling (DIA-NN), microtubule and ciliary functions (MaxDIA), RNA processing and splicing (Spectronaut), and ATP-dependent processes (MSFragger-DIA) (Figure 7C–D).

Distinct enrichment patterns were most evident in tissue data sets, which generally contained larger numbers of tool-specific proteins and significant enrichments. In contrast, blood-based data sets often yielded fewer tool-specific proteins and enriched terms, limiting the ability to assess biological differences between tools (Supplemental Figures S5–S6).

Collectively, these findings indicate that substantial fractions of the quantifiable proteome remain tool-specific and that these proteins can highlight distinct biological processes in certain data sets. Thus, reliance on a single processing tool may overlook potentially relevant biological information, providing a rationale for the subsequent evaluation of multitool differential expression integration while restricting analyses to outputs passing stringent FDR (1%) filtering to minimize the influence of lower-confidence identifications.

### Integrated Differential Expression Results

For each data set, the processing tool yielding the highest number of quantifiable proteins was designated as the main result for DEA. This included FragPipe for all label-free DDA data sets except the two vascular matrisome data sets, for which PD_SHT provided the highest quantifiable protein numbers (Figure 2). Among DIA data sets, Spectronaut was selected for two cardiac tissue data sets and one serum data set, while DIA-NN was selected for the plasma data set (Figure 3). The PQ500-spiked plasma data set was treated as an exception, with Spectronaut designated as the main result because of its dedicated support for PQ500 data processing. Selection of additional tools was based on the predefined agreement criteria, with pairwise abundance correlations and directional concordance rates summarized in Supplemental Figures S7–S8.

Sensitivity analyses demonstrated that tool inclusion was unaffected by increasing the correlation threshold from ρ > 0.4 to ρ > 0.5. In contrast, increasing the directional concordance requirement from 70% to 80% reduced the number of eligible additional tools in several data sets, particularly among label-free DDA analyses, while having little effect in some DIA data sets (Supplemental Figures S9–S10). These findings support the selected criteria as a practical balance between retaining complementary tool outputs and maintaining consistency with the main results.

Integration of complementary tool-specific proteins increased quantitative proteome coverage available for DEA by a median of 4.2% across label-free DDA data sets and 8.7% across DIA data sets (Supplemental Table S13). The impact of this additional protein coverage on DEP detection varied among data sets.

Across label-free DDA data sets, integration increased DEP detection by 3.3% (31 additional DEPs beyond the 943 identified by the main tool) in the cardiac proteome data set (PXD053721, 48 advanced heart failure patients versus 16 matched nonfailing controls; Figure 8A), by 25% (1 additional DEP beyond the 4 identified by the main tool) in a second cardiac proteome data set (PXD060431, 10 heart failure with preserved ejection fraction patients versus 10 nonfailing controls; Supplemental Figure S11A), and by 6.1% (29 additional DEPs beyond the 474 identified by the main tool) in the vascular proteome data set (PXD035563, 24 atherosclerotic versus 12 paired nondiseased regions; Figure 8B). No additional DEPs were identified from complementary tool outputs in the vascular matrisome data sets or the serum proteome data set.

**Figure 8:**
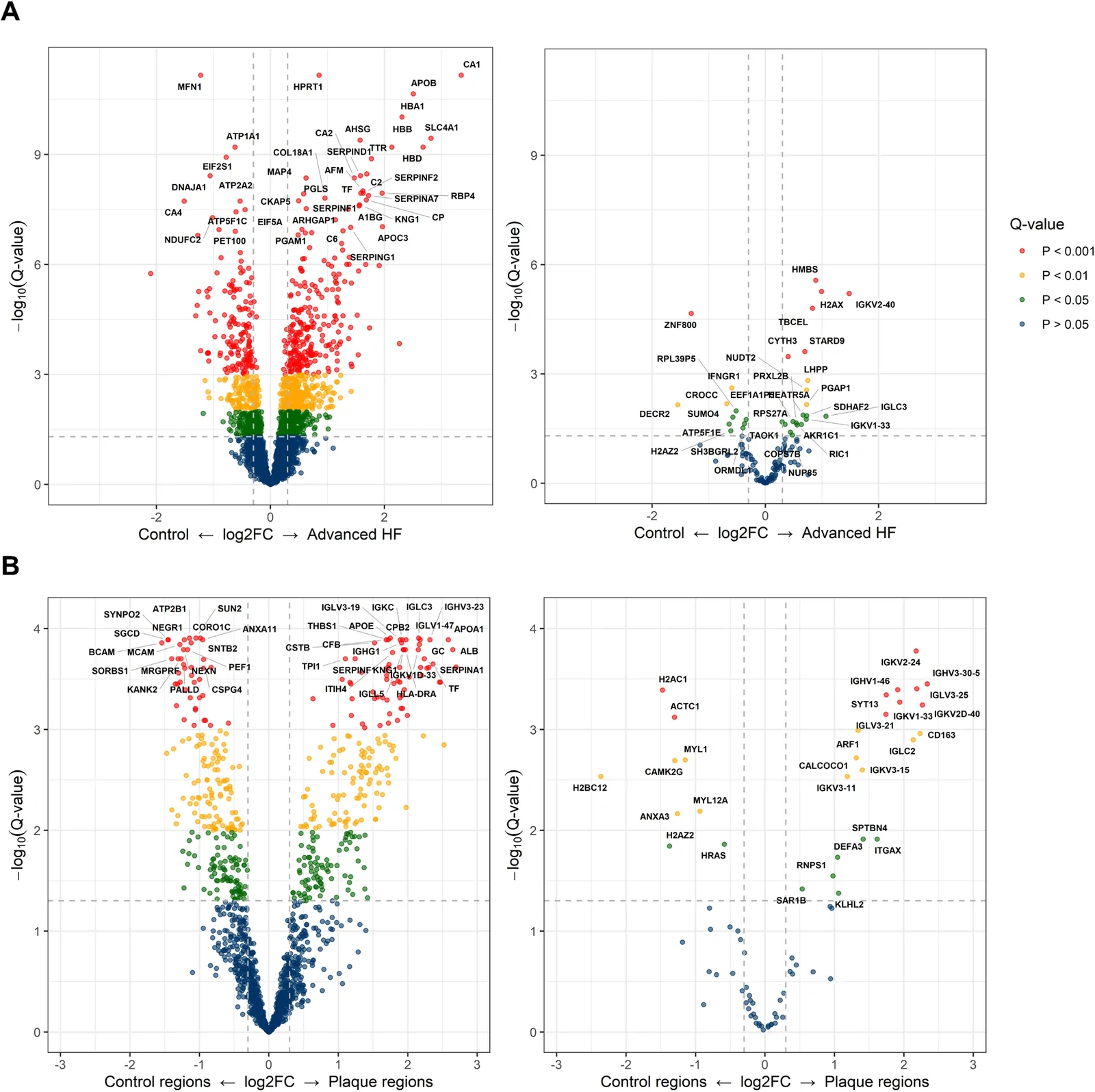
Volcano plots of differentially expressed proteins in label-free DDA data sets. Plots show log2 fold change versus -log10 Q-value, with the top 40 significant proteins labeled. Left panels show the main differential expression results and right panels show complementary results. Comparisons include (A) 48 advanced heart failure patients versus 16 nonfailing controls (cardiac proteome, PXD053721) and (B) 24 atherosclerotic versus 12 paired nondiseased regions (vascular proteome, PXD035563).

Across DIA data sets, integration increased DEP detection by 18.5% (5 additional DEPs beyond the 27 identified by the main tool) in the cardiac proteome data set (PXD018678, 15 ischemic cardiomyopathy patients versus 15 matched donor controls; Figure 9A), by 10.5% (2 additional DEPs beyond the 19 identified by the main tool) in the second cardiac proteome data set (PXD050610, 13 older versus 12 younger donors; Figure 9B), by 19.6% (37 additional DEPs beyond the 189 identified by the main tool) in the serum proteome data set (PXD025462, 25 MIS-C patients versus 24 healthy controls; Supplemental Figure S11B), and by 180% (18 additional DEPs beyond the 10 identified by the main tool) in the PQ500-spiked plasma data set (PXD024089, 48 COVID-19 ICU patients versus 11 sepsis ICU patients; Supplemental Figure S11C). In the plasma proteome data set (PXD047901, 8 severe COVID-19 patients versus 4 respiratory failure controls), integration identified one DEP that was not detected in the main analysis alone (Supplemental Figure S11D).

**Figure 9:**
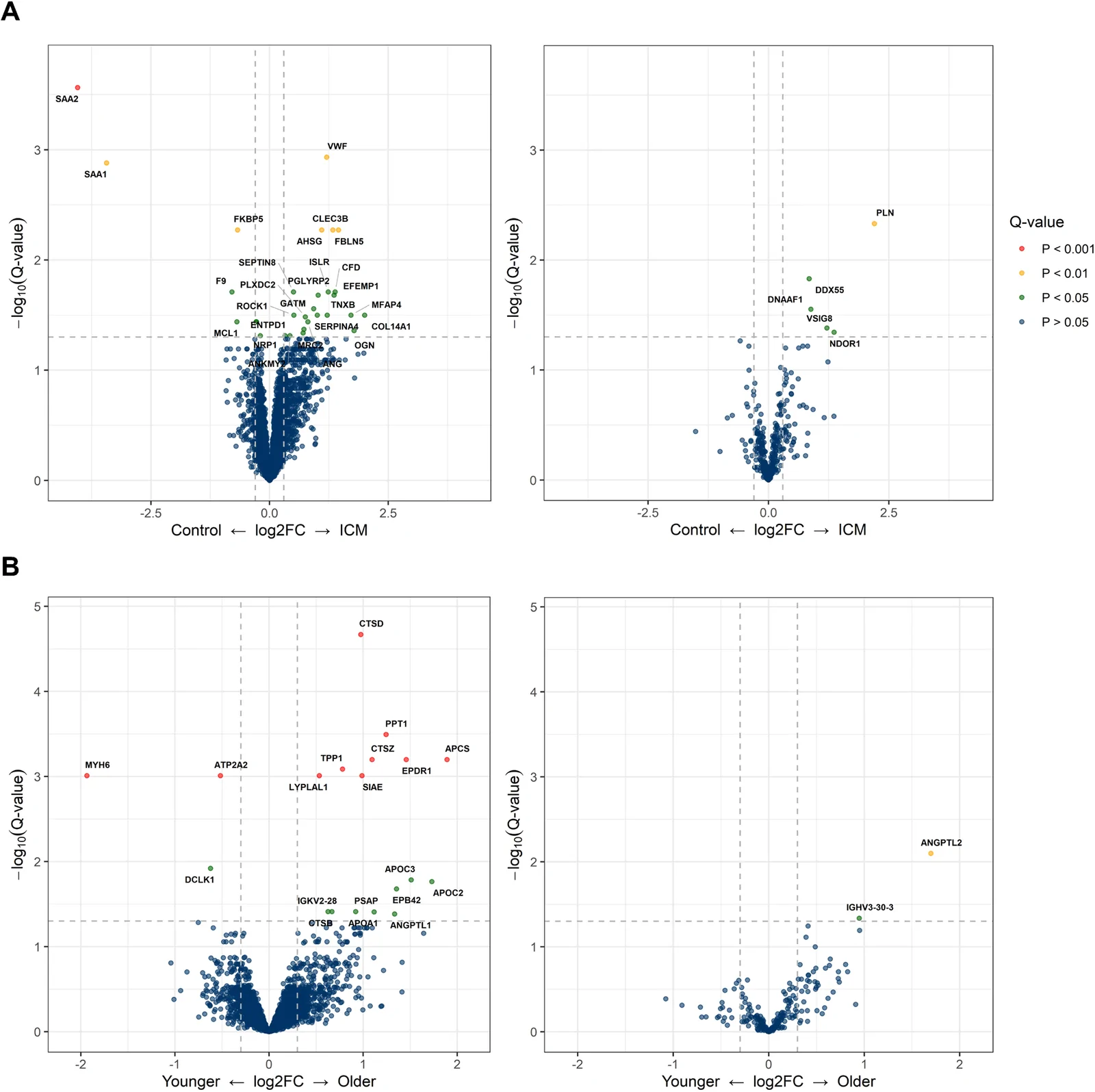
Volcano plots of differentially expressed proteins in DIA data sets. Plots show log2 fold change versus -log10 Q-value, with the top 40 significant proteins labeled. Left panels show the main differential expression results and right panels show complementary results. Comparisons include (A) 15 ischemic cardiomyopathy patients versus 15 matched donor controls (cardiac proteome, PXD018678) and (B) 13 older versus 12 younger donors (cardiac proteome, PXD050610).

Some newly recovered DEPs were supported by existing evidence linking them to the underlying disease pathology. For example, in the heart failure data set (PXD053721), H2AX is a key mediator of the DNA damage response, and increased γ-H2AX signaling has been associated with cardiomyocyte injury, maladaptive remodeling, and heart failure progression.^51^ Similarly, IFNGR1 participates in IFN-γ-mediated signaling pathways involved in cardiac hypertrophy and remodeling.^52^

In the atherosclerosis data set (PXD035563), CD163 has been associated with vulnerable carotid plaques and symptomatic disease, with elevated expression observed in plaques exhibiting features of instability and increased inflammatory activity.^53^ Likewise, ITGAX has been linked to macrophage-driven plaque inflammation and atherosclerotic progression,^54^ whereas ANXA3 has been associated with monocyte/macrophage-mediated inflammatory responses in cardiovascular disease.^55^

The recovery of these disease-relevant proteins highlights the potential of the integrative strategy to improve biological insight beyond that obtained from single-tool analyses alone. The full lists of additional significant DEPs identified through the integrative approach are provided in Supplemental Tables S14–S21.

### Study Limitations and Future Directions

Several limitations should be considered when interpreting the results of this comparative evaluation of proteomics data processing tools. First, the number of eligible cardiovascular proteomics data sets was limited by the study inclusion criteria, particularly for vascular tissue data sets. Consequently, some proteome subsets were represented by only a small number of data sets. Nevertheless, the included data sets consistently suggested that tool performance may vary across distinct proteome subsets, indicating that a single processing tool may not be optimal for all cardiovascular proteomics applications.

Second, all data sets analyzed in this study were generated using Orbitrap-based mass spectrometry platforms spanning multiple generations of instrument development, from LTQ Orbitrap XL to Orbitrap Exploris systems. Although Orbitrap instruments remain among the most widely used technologies in proteomics because of their high mass accuracy, sensitivity, and resolution,^1^ the observed tool performance may not fully generalize to newer or different instrument platforms. For example, Orbitrap Astral systems substantially increase MS/MS acquisition speed and proteome depth,^56^ whereas time-of-flight (TOF)-based instruments such as timsTOF incorporate ion mobility separation and generate data with acquisition characteristics that differ from conventional Orbitrap workflows.^57^ These technological differences may influence peptide identification, quantification, and protein inference performance, potentially altering the relative performance of data processing tools.

Third, because only real-world biological data sets were analyzed, no ground truth was available for direct estimation of false-positive and false-negative identification rates. To mitigate this limitation, stringent FDR thresholds (<1%) were applied at all available levels across tools. This approach was intended to reduce false-positive identifications while limiting losses in proteome coverage, as previously demonstrated for Spectronaut.^58^

Finally, although all evaluated tools provide extensive parameter customization, exhaustive per-tool optimization was not performed. Optimal parameter settings are often data set-specific and software-dependent, and extensive tuning in the absence of known ground truth may introduce analyst-driven variability while reducing comparability across tools. To promote fair comparisons while reflecting typical real-world usage as closely as possible, key parameters likely to influence results, including enzyme specificity, missed cleavages, modification settings, and precursor and fragment mass tolerances, were standardized whenever technically feasible.

As proteomics software continues to evolve and additional cardiovascular proteomics data sets become publicly available, further comparative analyses will be needed to evaluate newer tool versions and assess performance across emerging mass spectrometry technologies and diverse cardiovascular proteome subsets.

## Conclusions

In this study, we systematically compared multiple label-free DDA and DIA processing tools across cardiovascular tissue and blood-derived proteomics data sets. Tool performance varied across proteome subsets, although FragPipe and Spectronaut generally achieved the greatest quantitative proteome coverage for DDA and DIA analyses, respectively. Furthermore, integration of complementary tool outputs increased differential expression detection in several data sets. Together, these findings provide practical guidance for selecting proteomics data processing tools and support the use of integrative approaches to maximize biological insights from cardiovascular proteomics data.

## Supplementary Material



## Data Availability

All mass spectrometry proteomics data are publicly available through the ProteomeXchange Consortium via the PRIDE^59^ repository with the following data set identifiers: PXD005130, PXD008278, PXD018678, PXD024089, PXD035563, PXD047901, PXD050610, PXD053721, and PXD060431.

## Notes

Table S1: summary of LC-MS/MS data analysis workflows and bundled tools; Table S2: primary parameter settings used in the original analyses of label-free DDA data sets; Table S3: primary parameter settings used in the original analyses of DIA data sets; Table S4: tool performance metrics across label-free DDA and DIA data sets; Table S5: summary of DDA and DIA tool identification and quantification performance metrics across data sets; Table S6: peptide physicochemical properties by tool across six label-free DDA data sets and five DIA data sets; Table S7: molecular weight distributions of quantifiable proteins across label-free DDA tools; Table S8: molecular weight distributions of quantifiable proteins across DIA tools; Table S9: recovery rates and quantitative reproducibility of PQ500 heavy reference peptides in the PQ500-spiked plasma data set; Table S10: overlap of quantifiable proteins across processing tools and proportions of tool-specific proteins in each data set; Table S11: comparison of peptide support and identification confidence between shared and tool-specific proteins identified by label-free DDA tools; Table S12: comparison of peptide support and identification confidence between shared and tool-specific proteins identified by DIA tools; Table S13: changes in DEA input proteins and significant DEPs following multitool integration; Table S14: additional significantly dysregulated proteins identified in the cardiac proteome DDA data set (PXD053721); Table S15: additional significantly dysregulated proteins identified in the cardiac proteome DDA data set (PXD060431); Table S16: additional significantly dysregulated proteins identified in the vascular proteome DDA data set (PXD035563); Table S17: additional significantly dysregulated proteins identified in the cardiac proteome DIA data set (PXD018678); Table S18: additional significantly dysregulated proteins identified in the cardiac proteome DIA data set (PXD050610); Table S19: additional significantly dysregulated proteins identified in the serum proteome DIA data set (PXD025462); Table S20: additional significantly dysregulated proteins identified in the PQ500-spiked plasma proteome DIA data set (PXD024089); Table S21: additional significantly dysregulated proteins identified in the plasma proteome DIA data set (PXD047901); Figure S1: physicochemical characteristics of peptides identified by label-free DDA tools; Figure S2: physicochemical characteristics of peptides identified by DIA tools; Figure S3: molecular weight distributions of representative and quantifiable proteins across label-free DDA tools; Figure S4: molecular weight distributions of representative and quantifiable proteins across DIA tools; Figure S5: functional enrichment analysis of tool-specific proteins identified by label-free DDA tools; Figure S6: functional enrichment analysis of tool-specific proteins identified by DIA tools; Figure S7: protein abundance correlations and differential expression directional concordances across label-free DDA tools; Figure S8: protein abundance correlations and differential expression directional concordances across DIA tools; Figure S9: sensitivity analysis of tool inclusion across agreement thresholds in label-free DDA data sets; Figure S10: sensitivity analysis of tool inclusion across agreement thresholds in DIA data sets; Figure S11: volcano plots of differentially expressed proteins across label-free DDA and DIA data sets (PDF)

## Abbreviations

CEA: carotid endarterectomy
CV: coefficient of variation
DDA: data-dependent acquisition
DEA: differential expression analysis
DEP: differentially expressed protein
DIA: data-independent acquisition
FDR: false discovery rate
FE: fold enrichment
HF: heart failure
ICU: intensive care unit
LC-MS/MS: liquid chromatography-tandem mass spectrometry
log2FC: log2 fold change
MIS-C: multisystem inflammatory syndrome
MS: mass spectrometry
MW: molecular weight
PD: Proteome Discoverer
pI: isoelectric point
TPP: Trans-Proteomic Pipeline
