# Supplementary material for: Comparative Analysis of Data Processing Tools for Mass Spectrometry Proteomics in Cardiovascular Research
Source: J Proteome Res. 2026 Jul 31;25(11):5734–49. doi: 10.1021/acs.jproteome.6c00019 (PMC13750095; doi:10.1021/acs.jproteome.6c00019)
Supplement: Supplementary file 1 [file pr6c00019_si_001.pdf]

Supporting Information

Comparative Analysis of Data Processing Tools for Mass Spectrometry Proteomics in Cardiovascular Research

Thakorn Pruktanakul,[†,¶], Steven Lynham,[‡] Honglin Chen[§], Xiaoke Yin[§], Manuel Mayr[§], and Konstantinos Theofilatos*[,†]

[†]King's British Heart Foundation Centre, King’s College London, London SE5 9NU, UK

[‡]Proteomics Core Facility, King's College London, London SE5 9NU, UK

[§]National Heart and Lung Institute, Imperial College London, London W12 0BZ, UK

[¶]Division of Internal Medicine, Faculty of Medicine, Prince of Songkla University, Songkhla 90110, Thailand

*** Corresponding author**

E-mail: konstantinos.theofilatos@kcl.ac.uk

Table of Contents

Supplemental Tables

Table S1. Summary of LC-MS/MS data analysis workflows and bundled tools. 5

Table S2. Primary parameter settings used in the original analyses of label-free DDA datasets. .. 6

Table S3. Primary parameter settings used in the original analyses of DIA datasets. 7

Table S4. Tool performance metrics across label-free DDA and DIA datasets. 8

Table S5. Summary label-free DDA and DIA tool identification and quantification performance metrics across datasets. 10

Table S6. Peptide physicochemical properties by tool across 6 label-free DDA datasets and 4 DIA datasets. 13

Table S7. Molecular weight distribution of quantifiable proteins by dataset and label-free DDA tool... 17

Table S8. Molecular weight distribution of quantifiable proteins by dataset and DIA tool. 18

Table S9. Recovery rates and quantitative reproducibility of PQ500 heavy reference peptides in the PQ500-spiked plasma dataset (PXD024089)... 19

Table S10. Overlap of quantifiable proteins across processing tools and proportions of tool-specific proteins in each dataset. 20

Table S11. Comparison of peptide support and identification confidence between shared and tool-specific proteins identified by label-free DDA tools... 21

Table S12. Comparison of peptide support and identification confidence between shared and tool-specific proteins identified by DIA processing tools. 22

Table S13. Changes in DEA input proteins and significant DEPs following multi-tool integration. 33

Table S14. Additional significantly dysregulated proteins in the cardiac proteome DDA dataset (PXD053721). 36
Table S15. Additional significantly dysregulated proteins in the cardiac proteome DDA dataset (PXD060431). 36
Table S16. Additional significantly dysregulated proteins in the vascular proteome DDA dataset (PXD035563). 37
Table S17. Additional significantly dysregulated proteins in the cardiac proteome DIA dataset (PXD018678). 37
Table S18. Additional significantly dysregulated proteins in the cardiac proteome DIA dataset (PXD050610). 38
Table S19. Additional significantly dysregulated proteins in the serum proteome DIA dataset (PXD025462). 38
Table S20. Additional significantly dysregulated proteins in the PQ500-spiked plasma proteome DIA dataset (PXD024089). 39
Table S21. Additional significantly dysregulated proteins in the plasma proteome DIA dataset (PXD047901). 39

Supplemental Figures

Figure S1. Physicochemical characteristics of peptides identified by label-free DDA tools. 11
Figure S2. Physicochemical characteristics of peptides identified by DIA tools. 12
Figure S3. Molecular weight distribution of representative and quantifiable proteins across label-free DDA tools. 14
Figure S4. Molecular weight distribution of representative and quantifiable proteins across DIA tools. 16

Figure S5. Functional enrichment analysis of tool-specific proteins identified by different label-free DDA tools. 23

Figure S6. Functional enrichment analysis of tool-specific proteins identified by different DIA tools. 24

Figure S7. Protein abundance correlations and differential expression directional concordances across label-free DDA tools. 25

Figure S8. Protein abundance correlations and differential expression directional concordances across DIA tools. 27

Figure S9. Sensitivity analysis of tool inclusion across agreement thresholds in label-free DDA datasets. 29

Figure S10. Sensitivity analysis of tool inclusion across agreement thresholds in DIA datasets. 31

Figure S11. Volcano plots of differentially expressed proteins in label-free DDA and DIA datasets. 34

Table S1. Summary of LC-MS/MS data analysis workflows and bundled tools.

Software tool (workflow)	Version	Search engine	Validation tool or methodology	Quantification tool or mode
**DDA (label-free workflows)**				
MaxQuant (Standard)	2.7.5.0	Andromeda	Target-decoy FDR control	MaxLFQ
FragPipe (LFQ-MBR)	24.0	MSFragger v4.4	• MSBooster<br>• Percolator<br>• ProteinProphet	IonQuant v1.11.18
Proteome Discoverer (Label-free quantification)	3.2.0	Sequest HT	Percolator	Precursor ions quantifier node
**DIA (DDA-independent library workflows)**				
MaxQuant (MaxDIA, predicted mode)	2.7.5.0	Andromeda	Target-decoy FDR control	MaxLFQ
FragPipe (MSFragger-DIA)	24.0	MSFragger v4.4	• MSBooster<br>• Percolator<br>• ProteinProphet	Robust LC (high precision) in DIA-NN v1.8.2 beta 8
FragPipe (DIA-Umpire)				
DIA-NN (Library-free)	2.5.1	NN (cross-validated)	Target-decoy FDR control	QuantUMS (high precision)
Spectronaut (directDIA+, Deep)	20.3	Pulsar	Commercial	Automatic (sample size-dependent)

DDA, data-dependent acquisition; DIA, data-independent acquisition; FDR, false-discovery rate.

Table S2. Primary parameter settings used in the original analyses of label-free DDA datasets.

Accession No. (Citation)	Digestion enzyme	Missed cleavages	Variable/dynamic modifications	Fixed/static modifications	Maximum modifications	FDR (%)
PXD053721 (Beyhoff et al.[1])	Trypsin	3	- Oxidation of methionine<br>- N-terminal acetylation<br>- Deamidation of asparagine and glutamine	Carbamidomethylation of cysteine	5	1
PXD060431 (Jani et al.[2])	Trypsin	2	- Oxidation of methionine<br>- N-terminal acetylation	Carbamidomethylation of cysteine	3	1
PXD035563 (Blaser et al.[3])	Trypsin	4	- Oxidation of methionine<br>- N-terminal acetylation	Carbamidomethylation of cysteine	4	1
PXD005130 (Langley et al.[4])	Trypsin	2	- Oxidation of methionine, lysine, and proline	Carbamidomethylation of cysteine	3	1
PXD008278 (Bhosale et al.[5])	Trypsin	2	- Oxidation of methionine<br>- N-terminal acetylation	Methyl methanethiosulfonate of cysteine	3	1

DDA, data-dependent acquisition; FDR, false-discovery rate.

Table S3. Primary parameter settings used in the original analyses of DIA datasets.

Accession No. (reference)	Digestion enzyme	Missed cleavages	Variable/dynamic modifications	Fixed/static modifications	Maximum modifications	FDR (%)
PXD018678 (Li et al.[6])	Trypsin	2	- Oxidation of methionine<br>- N-terminal acetylation	Carbamidomethylation of cysteine	3	1
PXD050610 (Malecki et al.[7])	Trypsin	2	- Oxidation of methionine	Carbamidomethylation of cysteine	3	1
PXD024089 (Porritt et al.[8])	Trypsin	2	- Oxidation of methionine<br>- N-terminal acetylation	Carbamidomethylation of cysteine	3	1
PXD047901 (Ward et al.[9])	Trypsin	2	- Oxidation of methionine<br>- N-terminal acetylation	Carbamidomethylation of cysteine	3	1
PXD024089 (Gutmann et al.[10])	Trypsin	2	- Oxidation of methionine	Carbamidomethylation of cysteine	3	1

DIA, data-independent acquisition; FDR, false-discovery rate.

Table S4. Tool performance metrics across label-free DDA and DIA datasets.

Tool	Identification depth		Quantification quality		Tool-specific protein counts	Total runtime (hours)	Output file size (GB)
	Stripped peptide counts	Representative protein counts	Quantifiable protein counts	Missing values (%)			
**Cardiac tissue (PXD053721; Beyhoff et al.[1])**							
FragPipe	66,480	7,089	3,894	38.41	715	5.5	26.6
MaxQuant	36,048	4,505	3,024	26.2	100	40.1	375
PD (Sequest HT)	49,368	6,005	2,745	46.9	27	NA	375
**Cardiac tissue (PXD060431; Jani et al.[2])**							
FragPipe	60,096	5,878	1,748	51.68	271	1.1	9.71
MaxQuant	40,400	4,368	1,679	43.63	213	4.8	113
PD (Sequest HT)	48,823	5,305	891	66.54	3	NA	66.7
**Carotid plaque tissue (PXD035563; Blaser et al.[3])**							
FragPipe	28,423	2,897	1,453	36.73	352	1.2	5.1
MaxQuant	20,108	2,202	1,144	34.27	71	6.7	137
PD (Sequest HT)	8,187	1,395	184	69.82	2	NA	75.1
**Carotid plaque tissue, GuHCl-extracted (PXD005130; Langley et al.[4])**							
FragPipe	9,347	849	630	18.64	19	5.4	8.2
MaxQuant	7,899	934	347	47.98	18	12.9	109
PD (Sequest HT)	10,790	1,690	1,155	24.05	517	NA	78.4
**Carotid plaque tissue, NaHCl-extracted (PXD005130; Langley et al.[4])**							
FragPipe	10,829	1,093	956	9.14	27	5.4	8.2
MaxQuant	9,511	1,279	663	36.39	23	12.9	109
PD (Sequest HT)	11,015	1,848	1,512	12.27	539	NA	78.4
**Serum (PXD008278; Bhosale et al.[5])**							
FragPipe	5,946	316	198	31.1	1	1.4	3.7
MaxQuant	3,478	276	192	23.38	33	8.2	74.4
PD (Sequest HT)	3,922	323	148	48.13	3	NA	75.5

Table S4 (continued).

Tool	Identification depth		Quantification quality		Tool-specific protein counts	Total runtime (hours)	Output file size (GB)
	Stripped peptide counts	Representative protein counts	Quantifiable protein counts	Missing values (%)			
**Cardiac tissue (PXD018678; Li et al.[6])**							
DIA-NN	44,456	4,647	4,028	21.71	204	14.3	2.0
DIA-Umpire	28,053	3,005	2,914	2.6	38	12.7	54.7
MaxDIA	34,720	3,037	2,984	1.99	85	35.0	60.6
MSFragger-DIA	32,953	3,536	3,368	3.9	37	6.45	13.9
Spectronaut	48,883	4,353	4,165	3.31	303	NA	6.8
**Cardiac tissue (PXD050610; Malecki et al.[7])**							
DIA-NN	31,110	3,666	3,249	9.32	90	10.8	2.1
DIA-Umpire	18,303	2,316	2,268	2.04	34	10.9	51.3
MaxDIA	18,475	2,288	2,183	4.58	74	95.2	53.0
MSFragger-DIA	23,296	2,947	2,827	3.49	34	3.7	7.5
Spectronaut	35,720	3,818	3,652	3.88	429	NA	3.3
**Serum (PXD024089; Porritt et al.[8])**							
DIA-NN	6,523	505	320	28.8	18	7.6	1.7
DIA-Umpire	4,050	210	195	5.38	2	3.3	8.1
MaxDIA	3,379	317	219	21.93	12	11.7	22.6
MSFragger-DIA	6,279	490	383	15.77	58	3.8	6.0
Spectronaut	6,819	645	383	29.77	91	NA	2.3
**Plasma (PXD047901; Ward et al.[9])**							
DIA-NN	10,171	1,497	728	54.34	44	5.8	1.7
DIA-Umpire	4,595	480	442	5.76	11	3.5	12.7
MaxDIA	6,187	912	618	21.13	63	8.9	18.7
MSFragger-DIA	6,147	896	707	14.11	63	2.4	1.5
Spectronaut	8,356	1,326	685	32.25	102	NA	1.9

NA, not applicable due to the use of a different workstation.

Table S5. Summary label-free DDA and DIA tool identification and quantification performance metrics across datasets.

Tool	Identification depth		Quantification quality	
	**Stripped peptide counts, Median (Q1, Q3)**	**Representative protein counts, Median (Q1, Q3)**	**Quantifiable protein counts, Median (Q1, Q3)**	**% Missing values, Median (Q1, Q3)**
**Overall median across label-free DDA datasets (excluding the PQ500-spiked plasma dataset)**				
FragPipe	19,626 (9,718, 52,178)	1,995 (910, 5,133)	1,205 (712, 1,674)	33.9 (21.8, 38.0)
MaxQuant	14,810 (8,302, 32,063)	1,741 (1,020, 3,827)	904 (426, 1,545)	35.3 (28.2, 41.8)
PD (Sequest HT)	10,903 (8,838, 39,371)	1,769 (1,469, 4,441)	1,023 (361, 1,423)	47.5 (29.8, 61.9)
Friedman p-value	0.030	0.311	0.135	0.135
**Overall median across DIA datasets (excluding the PQ500-spiked plasma dataset)**				
DIA-NN	20,641 (9,259, 34,447)	2,582 (1,249, 3,911)	1,988 (626, 3,444)	25.26 (18.61, 35.19)
DIA-Umpire	11,449 (4,459, 20,741)	1,398 (412, 2,488)	1,355 (380, 2,430)	3.99 (2.46, 5.47)
MaxDIA	12,331 (5,485, 22,536)	1,600 (763, 2,475)	1,400 (518, 2,383)	12.86 (3.93, 21.33)
MSFragger-DIA	14,788 (6,246, 25,710)	1,922 (794, 3,094)	1,767 (626, 2,962)	9.00 (3.80, 14.52)
Spectronaut	22,038 (7,972, 39,011)	2,572 (1,156, 3,952)	2,168 (610, 3,780)	16.82 (3.74, 30.39)

Friedman tests were performed across the six label-free DDA datasets with datasets treated as matched blocks. Statistical comparisons were not performed for DIA workflows because the limited number of comparative datasets (n = 4) provided insufficient power for meaningful between-tool comparisons.

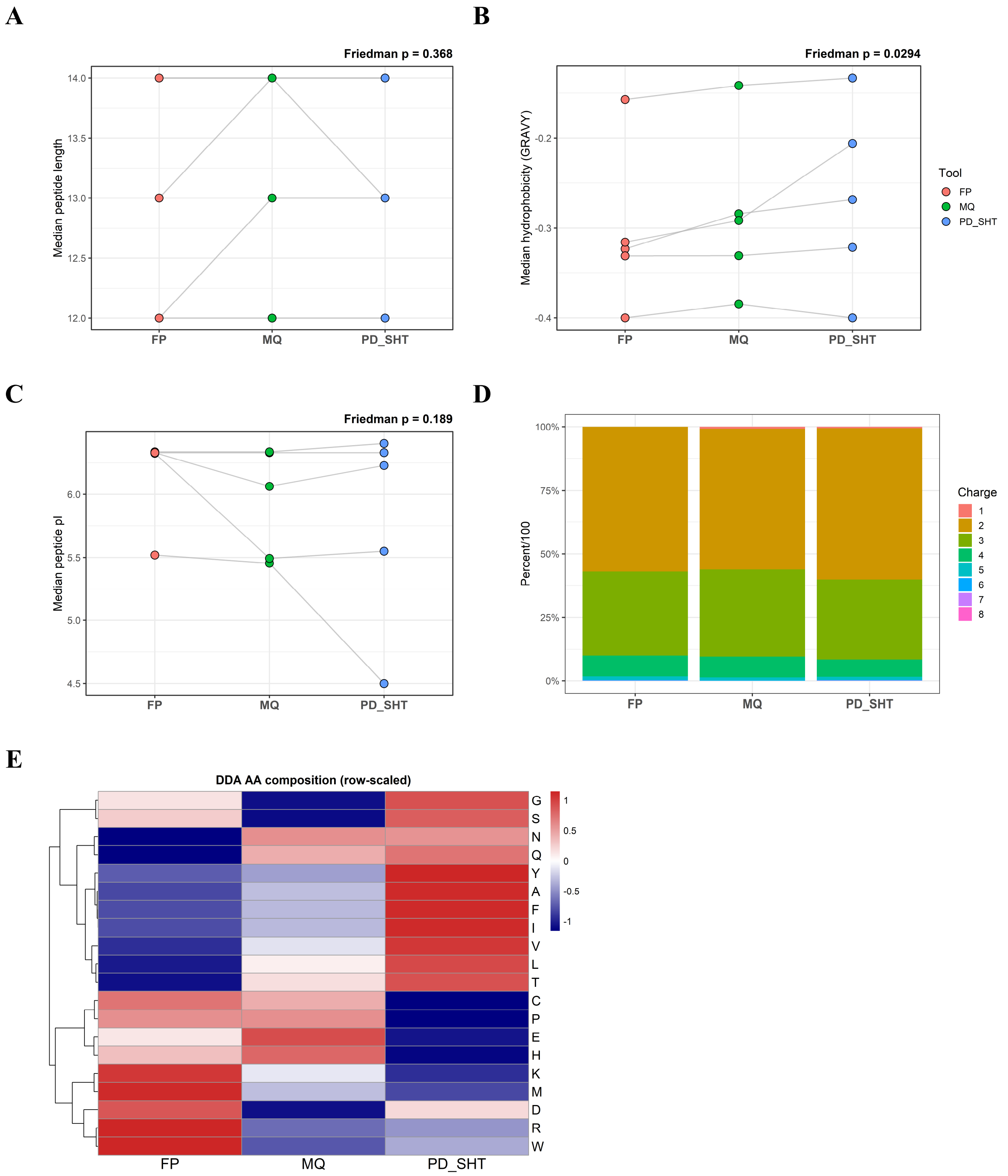

Figure S1. Physicochemical characteristics of peptides identified by label-free DDA tools. (A) Peptide length distribution. (B) Peptide hydrophobicity distribution calculated using the Kyte–Doolittle scale. (C) Peptide isoelectric point (pI) distribution. (D) Precursor charge-state distribution. (E) Amino acid composition of identified peptides. Distributions were calculated from all peptide identifications obtained from FragPipe (FP), MaxQuant (MQ), and Proteome Discoverer with Sequest HT (PD_SHT) across the evaluated label-free DDA datasets.

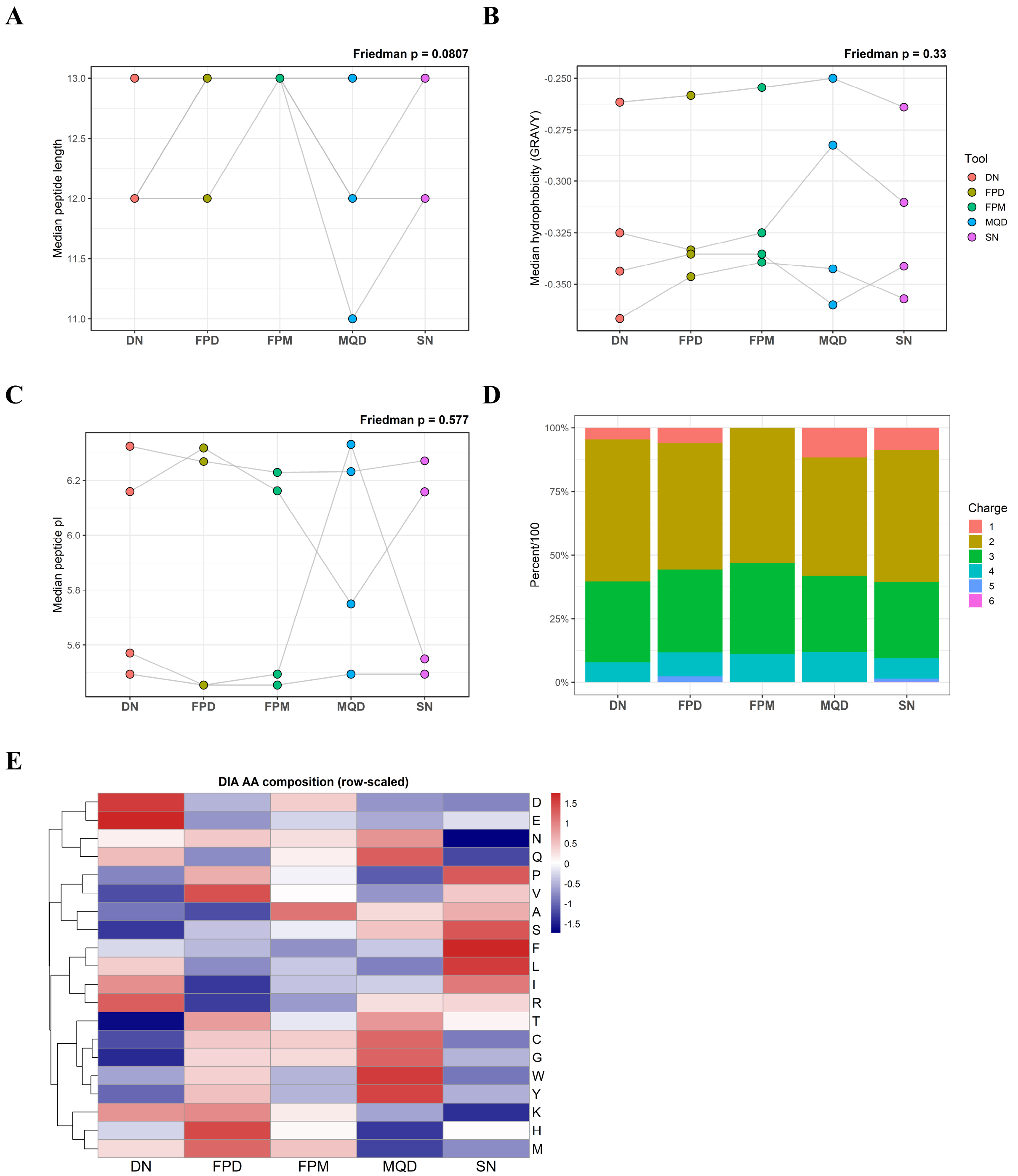

Figure S2. Physicochemical characteristics of peptides identified by DIA tools. (A) Peptide length distribution. (B) Peptide hydrophobicity distribution calculated using the Kyte–Doolittle scale. (C) Peptide isoelectric point (pI) distribution. (D) Precursor charge-state distribution. (E) Amino acid composition of identified peptides. Distributions were calculated from all peptide identifications obtained from DIA-NN (DN), DIA-Umpire (FPD), MSFragger-DIA (FPM), MaxDIA (MQD), and Spectronaut (SN) across the evaluated DIA datasets.

Table S6. Peptide physicochemical properties by tool across 6 label-free DDA datasets and 4 DIA datasets.

Tool	Identified peptides, median (Q1, Q3)	Length, median (Q1, Q3)	Hydrophobicity, median (Q1, Q3)	Isoelectric point, median (Q1, Q3)
**Label-free DDA**				
FragPipe	28,816 (11,385, 60,560)	13 (12, 14)	-0.32 (-0.33, -0.32)	6.33 (6.33, 6.33)
MaxQuant	22,377 (11,233, 39,138)	14 (13, 14)	-0.29 (-0.33, -0.28)	6.06 (5.49, 6.33)
PD (Sequest HT)	11,417 (8,593, 49,872)	13 (13, 14)	-0.27 (-0.32, -0.21)	6.23 (5.55, 6.33)
**DIA**				
DIA-NN	21,024 (9,561, 34,824)	12 (12, 12.2)	-0.33 (-0.35, -0.31)	5.86 (5.55, 6.20)
DIA-Umpire	7,211 (4,766, 18,585)	13 (13, 13)	-0.33 (-0.34, -0.26)	6.27 (5.45, 6.32)
MaxDIA	7,178 (6,075, 19,396)	12 (12, 13)	-0.28 (-0.34, -0.26)	6.23 (5.75, 6.33)
MSFragger-DIA	8,648 (6,394, 23,625)	13 (13, 13)	-0.32 (-0.34, -0.25)	6.16 (5.49, 6.23)
Spectronaut	9,031 (7,419, 36,079)	12 (12, 13)	-0.31 (-0.34, -0.26)	6.16 (5.55, 6.27)

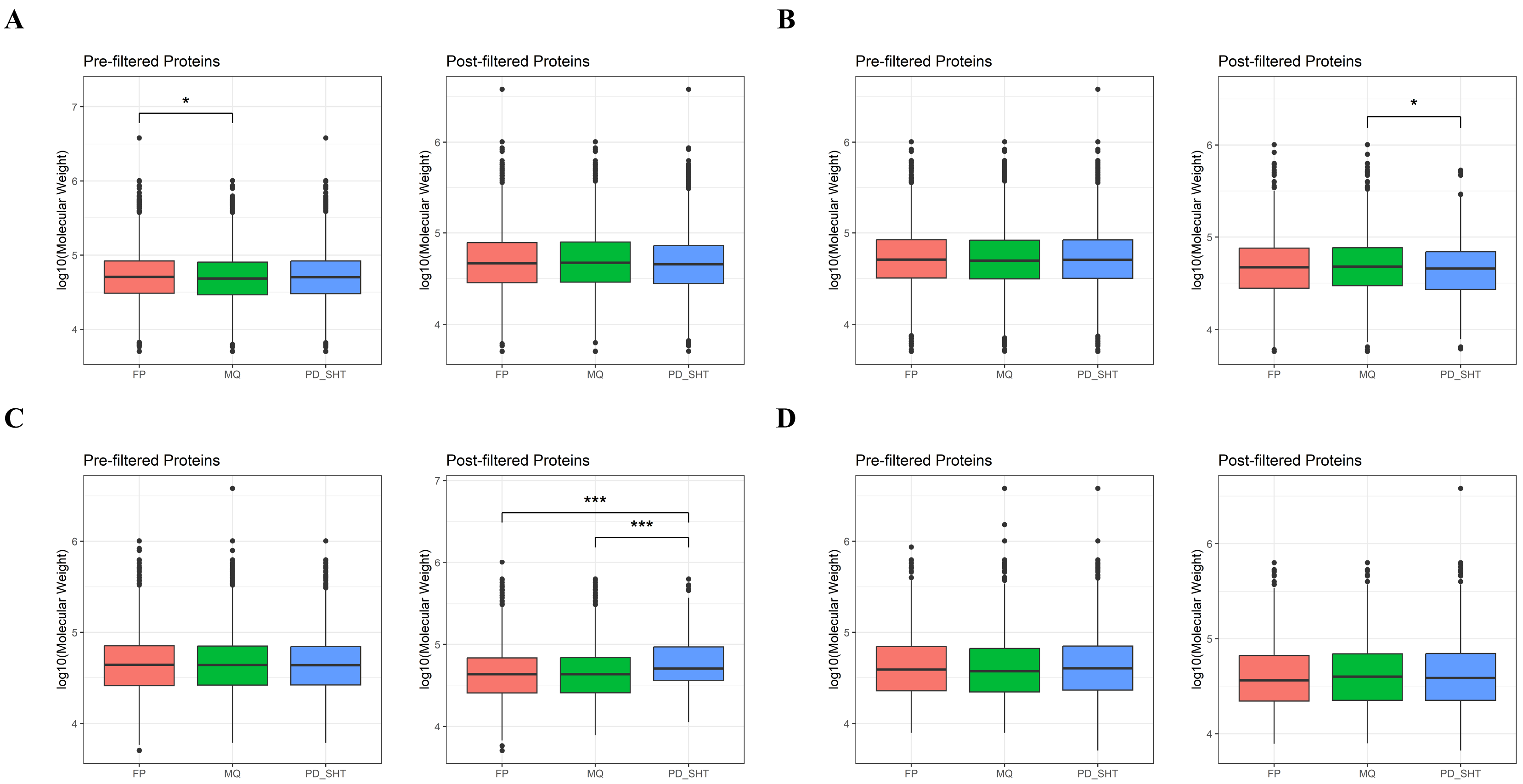

Figure S3. Molecular weight distribution of representative and quantifiable proteins across label-free DDA tools. Boxplots show log10-transformed protein molecular weights (Da) of representative (pre-filtered, left panels) and quantifiable (post-filtered, right panels) proteins identified by FragPipe (FP), MaxQuant (MQ), and Proteome Discoverer with Sequest HT (PD_SHT) in (A) cardiac proteome (PXD053721), (B) cardiac proteome (PXD060431), (C) vascular proteome (PXD035563), (D) core vascular matrisome (PXD005130), (E) soluble vascular matrisome (PXD005130), and (F) serum proteome (PXD008278). Statistical significance: *, $p < 0.05$; **, $p < 0.01$; ***, $p < 0.001$.

E **F**

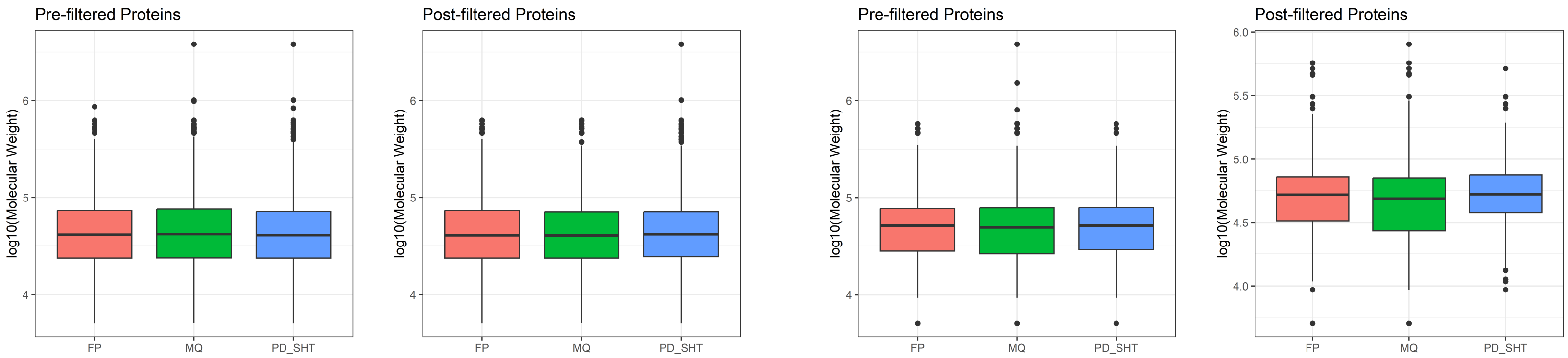

Figure S3 (continued). Molecular weight distribution of representative and quantifiable proteins across label-free DDA tools. Boxplots show log10-transformed protein molecular weights (Da) of representative (pre-filtered, left panels) and quantifiable (post-filtered, right panels) proteins identified by FragPipe (FP), MaxQuant (MQ), and Proteome Discoverer with Sequest HT (PD_SHT) in (A) cardiac proteome (PXD053721), (B) cardiac proteome (PXD060431), (C) vascular proteome (PXD035563), (D) core vascular matrisome (PXD005130), (E) soluble vascular matrisome (PXD005130), and (F) serum proteome (PXD008278). Statistical significance: *, $p < 0.05$; **, $p < 0.01$; ***, $p < 0.001$.

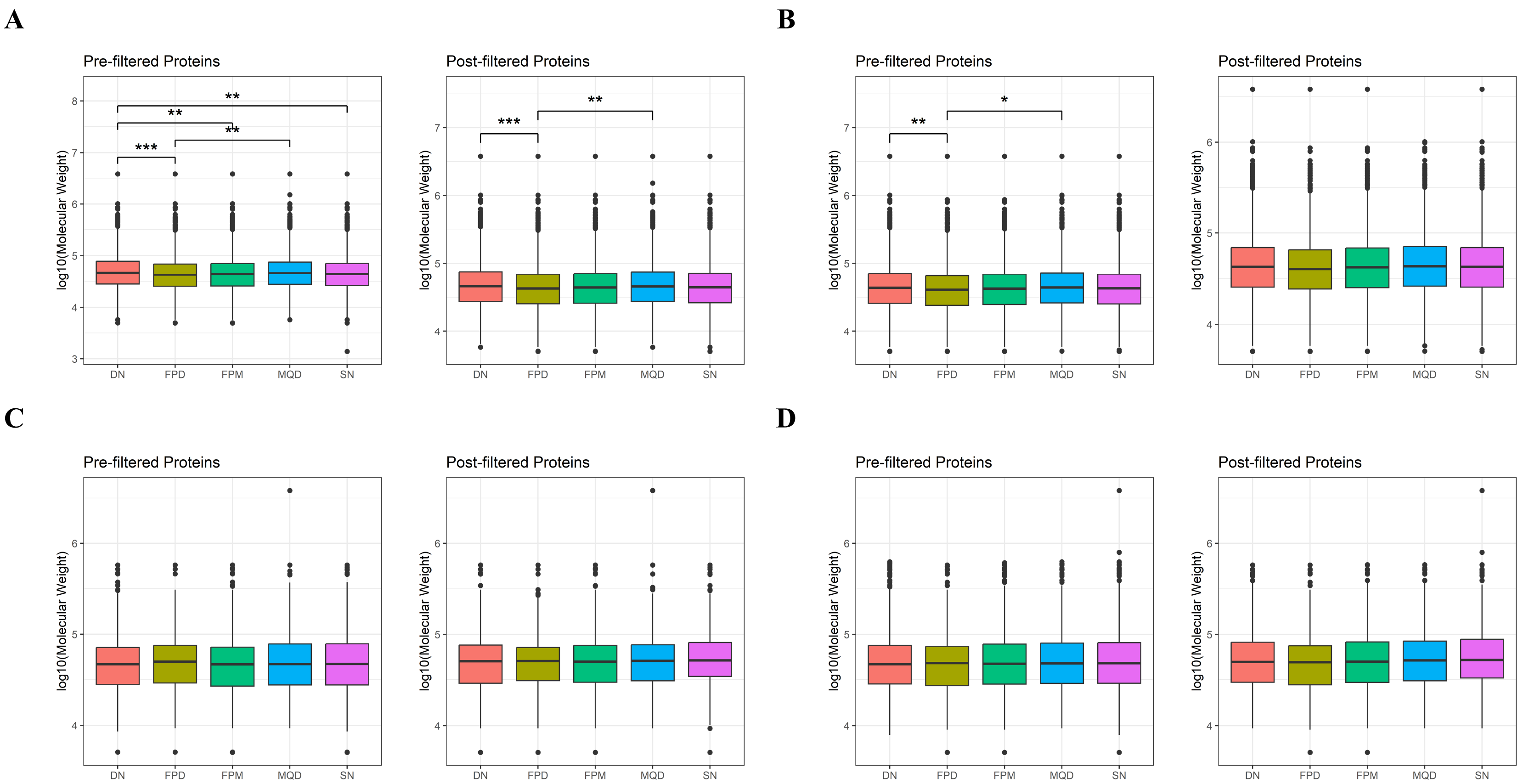

Figure S4. Molecular weight distribution of representative and quantifiable proteins across DIA tools.
Boxplots show log10-transformed protein molecular weights (Da) of representative (pre-filtered, left panels) and quantifiable (post-filtered, right panels) proteins identified by DIA-NN (DN), DIA-Umpire (FPD), MSFragger-DIA (FPM), MaxDIA (MQD), and Spectronaut (SN) in (A) cardiac proteome (PXD018678), (B) cardiac proteome (PXD050610), (C) serum proteome (PXD025462), and (D) plasma proteome (PXD047901). Statistical significance: *p < 0.05; **p < 0.01; ***p < 0.001.

Table S7. Molecular weight distribution of quantifiable proteins by dataset and label-free DDA tool.

Tool	Median (Q1, Q3), Da		Mean ± SD, Da	
	Representative proteins	Quantifiable proteins	Representative proteins	Quantifiable proteins
**Cardiac tissue (PXD053721, Beyhoff et al.[1])**				
FragPipe	50,433 (30,608, 83,542)	46,744 (28,441, 78,504)	69,403 ± 81,161	66,690 ± 91,108
MaxQuant	48,139 (29,174, 80,859)	47,442 (28,807, 79,456)	67,853 ± 70,315	67,255 ± 72,911
PD (Sequest HT)	50,196 (30,189, 83,435)	45,566 (27,873, 72,849)	69,767 ± 84,998	65,140 ± 98,893
**Cardiac tissue (PXD060431; Jani et al.[2])**				
FragPipe	51,255 (32,006, 84,100)	47,355 (27,844, 75,776)	69,011 ± 64,487	64,045 ± 67,707
MaxQuant	50,143 (31,306, 83,240)	48,317 (29,677, 76,741)	68,550 ± 67,284	65,451 ± 68,422
PD (Sequest HT)	51,103 (31,785, 83,786)	45,862 (27,079, 69,603)	69,775 ± 83,315	58,468 ± 54,436
**Carotid plaque tissue (PXD035563, Blaser et al.[3])**				
FragPipe	44,231 (25,835, 71,224)	42,964 (25,511, 68,526)	61,346 ± 66,441	61,373 ± 70,579
MaxQuant	43,974 (26,183, 70,797)	42,916 (25,518, 68,920)	64,016 ± 105,890	60,910 ± 66,725
PD (Sequest HT)	43,787 (26,311, 70,029)	50,975 (36,134, 93,252)	62,494 ± 71,145	87,883 ± 100,604
**Carotid plaque tissue, GuHCl-extracted (PXD005130, Langley et al.[4])**				
FragPipe	39,232 (22,678, 70,021)	36,414 (22,099, 66,678)	63,953 ± 82,647	60,202 ± 74,903
MaxQuant	37,478 (22,094, 66,546)	39,747 (22,490, 69,462)	66,958 ± 154,655	66,756 ± 84,169
PD (Sequest HT)	40,380 (23,090, 70,681)	38,467 (22,548, 69,922)	63,363 ± 116,267	63,807 ± 131,793
**Carotid plaque tissue, NaCl-extracted (PXD005130, Langley et al.[4])**				
FragPipe	41,390 (23,603, 73,243)	40,833 (23,593, 73,680)	62,892 ± 73,917	62,957 ± 72,258
MaxQuant	42,019 (23,689, 75,913)	40,669 (23,581, 71,009)	68,548 ± 133,345	62,395 ± 75,530
PD (Sequest HT)	41,002 (23,601, 71,363)	41,920 (24,426, 71,125)	63,820 ± 114,056	64,238 ± 120,579
**Serum (PXD008278, Bhosale et al.[5])**				
FragPipe	51,435 (27,887, 77,102)	52,391 (32,596, 72,920)	68,353 ± 73,076	69,310 ± 77,600
MaxQuant	49,282 (26,218, 78,330)	48,818 (27,182, 71,510)	90,790 ± 258,299	72,138 ± 97,096
PD (Sequest HT)	51,594 (28,980, 78,853)	52,760 (37,783, 75,654)	69,739 ± 72,477	66,396 ± 60,447

Table S8. Molecular weight distribution of quantifiable proteins by dataset and DIA tool.

Tool	Median (Q1, Q3), Da		Mean ± SD, Da	
	Representative proteins	Quantifiable proteins	Representative proteins	Quantifiable proteins
**Cardiac tissue (PXD018678, Li et al.[6])**				
DIA-NN	46,538 (27,948, 77,825)	45,885 (27,367, 75,019)	65,862 ± 87,476	64,766 ± 89,444
DIA-Umpire	42,718 (25,301, 68,772)	42,562 (25,383, 68,508)	61,577 ± 97,312	61,505 ± 98,137
MaxDIA	45,808 (27,614, 75,341)	45,547 (27,551, 74,818)	69,040 ± 107,988	67,691 ± 105,135
MSFragger-DIA	43,918 (25,690, 70,371)	43,984 (25,858, 70,677)	62,428 ± 93,072	62,533 ± 93,660
Spectronaut	43,946 (26,152, 71,028)	44,187 (26,249, 71,570)	61,502 ± 85,447	61,883 ± 86,815
**Cardiac tissue (PXD050610; Malecki et al.[7])**				
DIA-NN	43,588 (25,778, 70,776)	42,760 (25,428, 69,128)	62,345 ± 91,232	61,332 ± 94,218
DIA-Umpire	40,693 (24,028, 65,412)	40,607 (24,267, 65,369)	59,333 ± 101,630	59,317 ± 102,461
MaxDIA	43,918 (26,121, 72,193)	43,390 (26,051, 70,915)	67,150 ± 112,303	64,939 ± 79,848
MSFragger-DIA	42,293 (24,828, 68,809)	42,283 (25,063, 68,568)	61,243 ± 96,486	61,369 ± 97,898
Spectronaut	42,730 (25,254, 68,891)	42,875 (25,425, 69,224)	60,641 ± 90,030	61,129 ± 91,507
**Serum (PXD024089, Porritt et al.[8])**				
DIA-NN	46,987 (27,745, 71,540)	50,867 (28,862, 76,136)	64,106 ± 66,513	67,235 ± 70,446
DIA-Umpire	50,143 (28,964, 75,414)	50,992 (30,746, 71,774)	69,615 ± 77,022	68,770 ± 76,675
MaxDIA	47,173 (27,566, 78,182)	51,276 (30,688, 76,684)	82,663 ± 224,197	86,154 ± 263,970
MSFragger-DIA	46,862 (26,763, 71,896)	50,229 (29,557, 75,702)	64,518 ± 70,555	68,419 ± 73,603
Spectronaut	47,371 (27,566, 78,287)	51,924 (34,259, 81,276)	67,808 ± 74,273	73,197 ± 80,434
**Plasma (PXD047901, Ward et al.[9])**				
DIA-NN	47,173 (28,357, 75,702)	49,924 (29,556, 81,745)	66,156 ± 69,486	69,514 ± 69,750
DIA-Umpire	48,424 (27,201, 73,701)	49,525 (27,700, 74,792)	67,669 ± 75,038	67,758 ± 73,067
MaxDIA	48,142 (28,764, 80,057)	51,833 (30,680, 84,057)	70,833 ± 79,155	74,778 ± 80,095
MSFragger-DIA	47,684 (28,241, 78,208)	50,254 (29,482, 82,500)	68,024 ± 72,123	70,197 ± 70,931
Spectronaut	48,396 (28,837, 80,858)	52,442 (32,949, 88,223)	73,828 ± 129,613	81,809 ± 165,177

Table S9. Recovery rates and quantitative reproducibility of PQ500 heavy reference peptides in the PQ500-spiked plasma dataset (PXD024089).

Tool	PQ500 peptides detected (n)	Recovery (%)	Median CV (%)	IQR CV (%)	Peptides included in CV calculation (n)
DIA-NN	720	89.6	15.0	7.7	717
DIA-Umpire	757	94.2	22.4	6.7	757
MaxDIA	677	84.2	52.9	37.7	671
MSFragger-DIA	750	93.3	22.3	7.1	747
Spectronaut	679	84.5	13.3	10.4	679

Recovery (%) was calculated as the percentage of the 804 PQ500 heavy reference peptides detected after tool-specific confidence filtering. Quantitative reproducibility was assessed using the coefficient of variation (CV) of peptide abundances across samples. Median and interquartile range (IQR) values were calculated across recovered peptides. Peptides with insufficient observations for CV calculation were excluded.

Table S10. Overlap of quantifiable proteins across processing tools and proportions of tool-specific proteins in each dataset.

Dataset	Tools compared (n)	Quantifiable protein union (n)	Shared proteins (n, %)	Tool-specific proteins (n, %)
**Label-free DDA**				
Cardiac proteome (PXD053721)	3	4,028	3,186 (79.1)	842 (20.9)
Cardiac proteome (PXD060431)	3	1,971	1,484 (75.29)	487 (24.71)
Vascular proteome (PXD035563)	3	1,527	1,102 (72.17)	425 (27.83)
Core vascular matrisome (PXD005130)	3	1,196	642 (53.68)	554 (46.32)
Soluble vascular matrisome (PXD005130)	3	1,567	978 (62.41)	589 (37.59)
Serum proteome (PXD008278)	3	233	196 (84.12)	37 (15.88)
**DIA**				
Cardiac proteome (PXD018678)	5	4,622	3,955 (85.57)	667 (14.43)
Cardiac proteome (PXD050610)	5	3,955	3,294 (83.29)	661 (16.71)
Serum proteome (PXD024089)	5	509	328 (64.44)	181 (35.56)
Plasma proteome (PXD047901)	5	1,015	732 (72.12)	283 (27.88)

Table S11. Comparison of peptide support and identification confidence between shared and tool-specific proteins identified by label-free DDA tools.

Tool	Shared proteins			Tool-specific proteins		
	n	Unique peptides, median (Q1, Q3)	Confidence metric*, median (Q1, Q3)	n	Unique peptides, median (Q1, Q3)	Confidence metric*, median (Q1, Q3)
**Cardiac tissue (PXD053721; Beyhoff et al.[1])**						
FragPipe	6,374	6.0 (3.0, 12.0)	1.0 (1.0, 1.0)	715	6.0 (4.0, 9.0)	1.0 (1.0, 1.0)
MaxQuant	4,405	5.0 (2.0, 9.0)	21.8 (3.7, 142.5)	100	2.0 (1.0, 3.0)	7.5 (1.9, 42.5)
PD (Sequest HT)	5,978	4.0 (2.0, 9.0)	256.9 (43.6, 1125.2)	27	2.0 (1.0, 4.0)	236.6 (110.0, 567.6)
**Cardiac tissue (PXD060431; Jani et al.[2])**						
FragPipe	5,607	5.0 (2.0, 13.0)	1.0 (1.0, 1.0)	271	8.0 (6.0, 13.0)	1.0 (1.0, 1.0)
MaxQuant	4,155	5.0 (2.0, 11.0)	11.3 (3.3, 49.6)	213	8.0 (5.0, 14.0)	29.8 (9.4, 83.9)
PD (Sequest HT)	5,302	4.0 (2.0, 10.0)	68.2 (17.8, 268.3)	3	2.0 (2.0, 25.0)	734.9 (467.6, 911.9)
**Carotid plaque tissue (PXD035563; Blaser et al.[3])**						
FragPipe	2,545	5.0 (2.0, 11.0)	1.0 (0.999, 1.0)	352	5.0 (3.0, 8.2)	1.0 (1.0, 1.0)
MaxQuant	2,131	4.0 (2.0, 9.0)	16.7 (5.0, 88.5)	71	2.0 (1.0, 4.0)	7.3 (2.8, 46.4)
PD (Sequest HT)	1,393	3.0 (1.0, 6.0)	0.0 (0.0, 0.0)	2	2.5 (2.2, 2.8)	11.8 (11.5, 12.1)
**Carotid plaque tissue, GuHCl-extracted (PXD005130; Langley et al.[4])**						
FragPipe	830	7.0 (4.0, 13.0)	1.0 (1.0, 1.0)	19	3.0 (2.0, 4.5)	1.0 (0.998, 1.0)
MaxQuant	916	4.0 (2.0, 9.0)	20.7 (6.1, 75.5)	18	2.0 (1.0, 3.0)	21.4 (3.8, 105.5)
PD (Sequest HT)	1,173	3.0 (2.0, 7.0)	161.3 (33.6, 811.1)	517	3.0 (2.0, 5.0)	93.5 (32.4, 256.8)
**Carotid plaque tissue, NaHCl-extracted (PXD005130; Langley et al.[4])**						
FragPipe	1,066	6.0 (4.0, 11.0)	1.0 (1.0, 1.0)	27	3.0 (2.0, 5.0)	1.0 (0.997, 1.0)
MaxQuant	1,256	4.0 (2.0, 8.0)	15.5 (5.4, 49.4)	23	2.0 (1.0, 3.0)	22.0 (7.8, 58.6)
PD (Sequest HT)	1,241	4.0 (2.0, 8.0)	224.3 (73.1, 823.5)	539	2.0 (1.0, 3.0)	30.3 (13.4, 69.7)
**Serum (PXD008278; Bhosale et al.[5])**						
FragPipe	313	8.0 (4.0, 22.0)	1.0 (1.0, 1.0)	3	7.0 (4.5, 13.5)	1.0 (1.0, 1.0)
MaxQuant	243	6.0 (2.5, 12.0)	46.6 (7.1, 242.4)	33	3.0 (2.0, 5.0)	6.8 (3.9, 18.1)
PD (Sequest HT)	322	5.0 (2.0, 11.0)	307.9 (41.4, 1745.6)	1	3.0 (3.0, 3.0)	695.0 (695.0, 695.0)

*Confidence metric corresponds to the native scoring system of each software package (Protein Probability for FragPipe, Andromeda Score for MaxQuant, and Sequest HT Score for Proteome Discoverer).

Table S12. Comparison of peptide support and identification confidence between shared and tool-specific proteins identified by DIA processing tools.

Tool	Shared proteins			Tool-specific proteins		
	n	Unique peptides, median (Q1, Q3)	Confidence metric*, median (Q1, Q3)	n	Unique peptides, median (Q1, Q3)	Confidence metric*, median (Q1, Q3)
**Cardiac tissue (PXD018678, Li et al.[6])**						
DIA-NN	4,443	6.0 (3.0, 11.0)	2.14E-04 (2.12E-04, 7.29E-04)	204	2.0 (1.5, 3.0)	9.74E-04 (4.83E-04, 1.94E-03)
DIA-Umpire	2,967	5.0 (2.0, 10.0)	4.36E-07 (1.45E-07, 2.24E-06)	38	1.0 (1.0, 1.0)	5.32E-06 (3.81E-07, 2.46E-05)
MaxDIA	2,952	7.0 (4.0, 12.0)	4.31E+01 (2.47E+01, 8.22E+01)	85	3.0 (2.0, 5.0)	2.21E+01 (1.42E+01, 2.84E+01)
MSFragger-DIA	3,499	5.0 (2.0, 10.0)	1.35E-06 (3.19E-07, 5.33E-06)	37	1.0 (1.0, 2.0)	1.11E-05 (1.20E-06, 1.22E-04)
Spectronaut	4,050	8.0 (4.0, 14.0)	7.55E-05 (2.19E-06, 5.31E-04)	303	2.0 (1.0, 3.0)	8.34E-04 (2.11E-04, 2.99E-03)
**Cardiac tissue (PXD050610; Malecki et al.[7])**						
DIA-NN	3,576	5.0 (2.0, 9.0)	1.10E-04 (5.93E-05, 5.22E-04)	90	2.0 (1.0, 2.0)	7.16E-04 (6.31E-05, 2.07E-03)
DIA-Umpire	2,282	4.0 (2.0, 8.0)	1.23E-06 (3.85E-07, 5.44E-06)	34	1.0 (1.0, 1.0)	5.88E-06 (7.73E-07, 3.99E-05)
MaxDIA	2,214	4.0 (3.0, 8.0)	2.93E+01 (1.76E+01, 5.41E+01)	74	3.0 (2.0, 4.0)	1.72E+01 (1.46E+01, 2.21E+01)
MSFragger-DIA	2,913	4.0 (2.0, 8.0)	1.64E-06 (4.09E-07, 6.73E-06)	34	1.0 (1.0, 2.0)	8.06E-06 (8.41E-07, 4.40E-05)
Spectronaut	3,389	6.0 (3.0, 12.0)	2.94E-05 (7.64E-07, 2.44E-04)	429	2.0 (1.0, 3.0)	4.57E-04 (9.94E-05, 1.19E-03)
**Serum (PXD024089, Porritt et al.[8])**						
DIA-NN	487	7.0 (3.0, 14.0)	2.97E-04 (2.08E-04, 5.48E-04)	18	2.0 (1.0, 2.0)	2.97E-04 (2.11E-04, 6.75E-04)
DIA-Umpire	208	10.5 (6.0, 23.2)	5.17E-07 (1.00E-09, 1.39E-05)	2	1.5 (1.2, 1.8)	4.97E-05 (2.61E-05, 7.33E-05)
MaxDIA	305	6.0 (3.0, 11.0)	3.87E+01 (1.97E+01, 7.95E+01)	12	3.0 (3.0, 6.0)	1.94E+01 (1.61E+01, 3.93E+01)
MSFragger-DIA	432	7.0 (3.0, 16.0)	2.63E-06 (1.00E-09, 1.46E-04)	58	3.0 (1.0, 4.0)	1.13E-04 (2.29E-05, 1.46E-04)
Spectronaut	554	6.0 (2.0, 13.0)	4.14E-04 (1.46E-07, 6.23E-03)	91	1.0 (1.0, 2.0)	4.99E-04 (4.45E-05, 1.20E-03)
**Plasma (PXD047901, Ward et al.[9])**						
DIA-NN	1,453	3.0 (2.0, 7.0)	5.28E-04 (5.28E-04, 1.53E-03)	44	2.0 (1.0, 3.0)	5.28E-04 (5.28E-04, 1.45E-03)
DIA-Umpire	469	5.0 (2.0, 11.0)	1.00E-09 (1.00E-09, 2.09E-05)	11	1.0 (1.0, 2.0)	9.36E-05 (1.57E-05, 2.60E-04)
MaxDIA	849	4.0 (2.0, 7.0)	2.37E+01 (1.36E+01, 4.87E+01)	63	3.0 (2.0, 3.5)	1.80E+01 (1.21E+01, 2.46E+01)
MSFragger-DIA	833	3.0 (2.0, 7.0)	2.24E-05 (8.27E-07, 2.23E-04)	63	1.0 (1.0, 2.0)	2.23E-04 (2.97E-05, 2.24E-04)
Spectronaut	1,224	4.0 (2.0, 7.0)	1.14E-03 (2.08E-04, 8.48E-03)	102	1.0 (1.0, 2.0)	1.52E-03 (2.70E-04, 3.70E-03)

*Confidence metric corresponds to the native confidence measure exported by each software package (q-value for DIA-NN, DIA-Umpire, MSFragger-DIA and Spectronaut; score for MaxDIA).

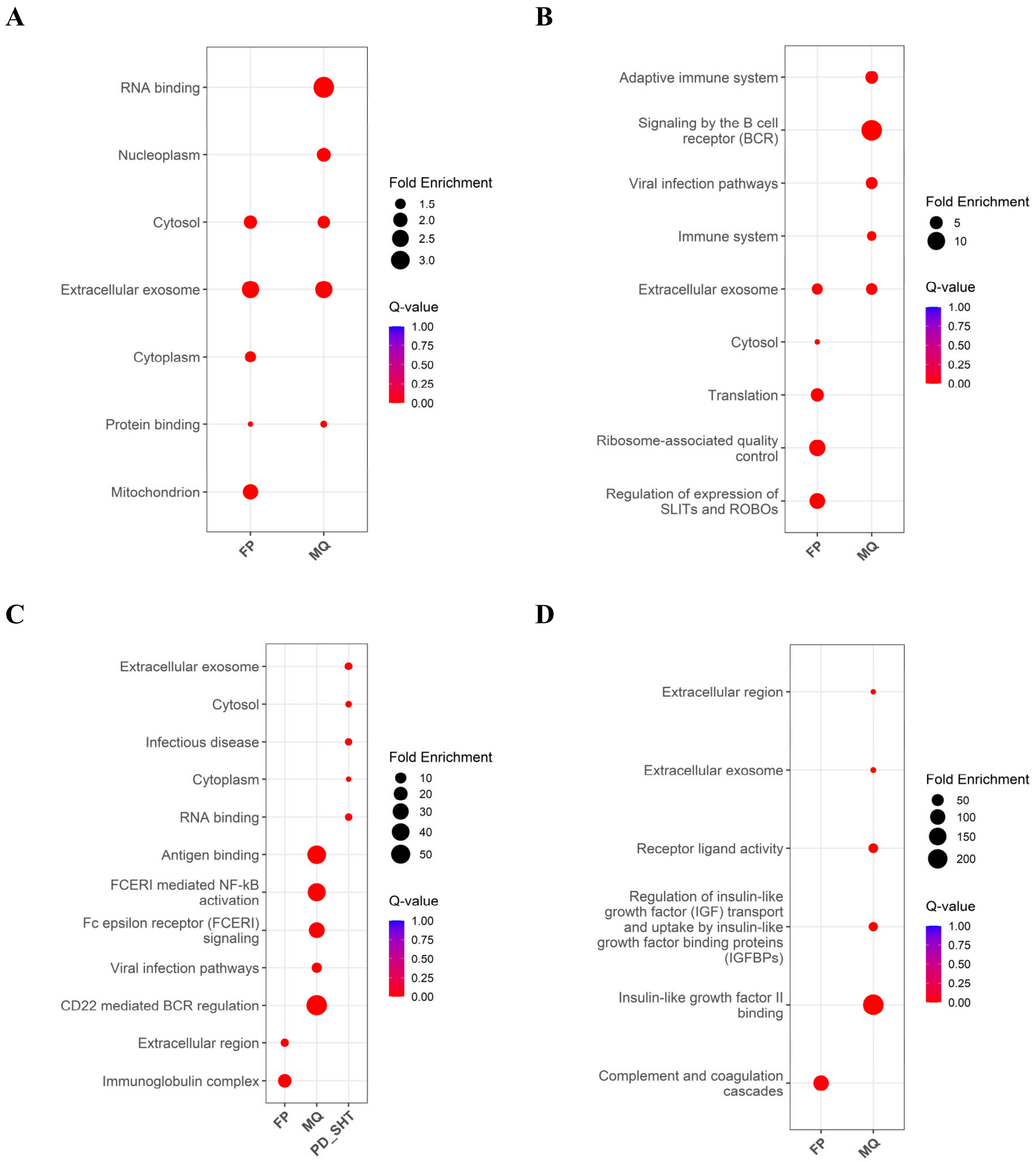

Figure S5. Functional enrichment analysis of tool-specific proteins identified by different label-free DDA tools.

The five most significantly enriched functional terms associated with tool-specific proteins are shown for label-free DDA tools, including FragPipe (FP), MaxQuant (MQ), and Proteome Discoverer with Sequest HT (PD_SHT) in (A) cardiac proteome (PXD060431), (B) vascular proteome (PXD035563), (C) soluble vascular matrisome (PXD005130), and (D) serum proteome (PXD008278).

A **B**

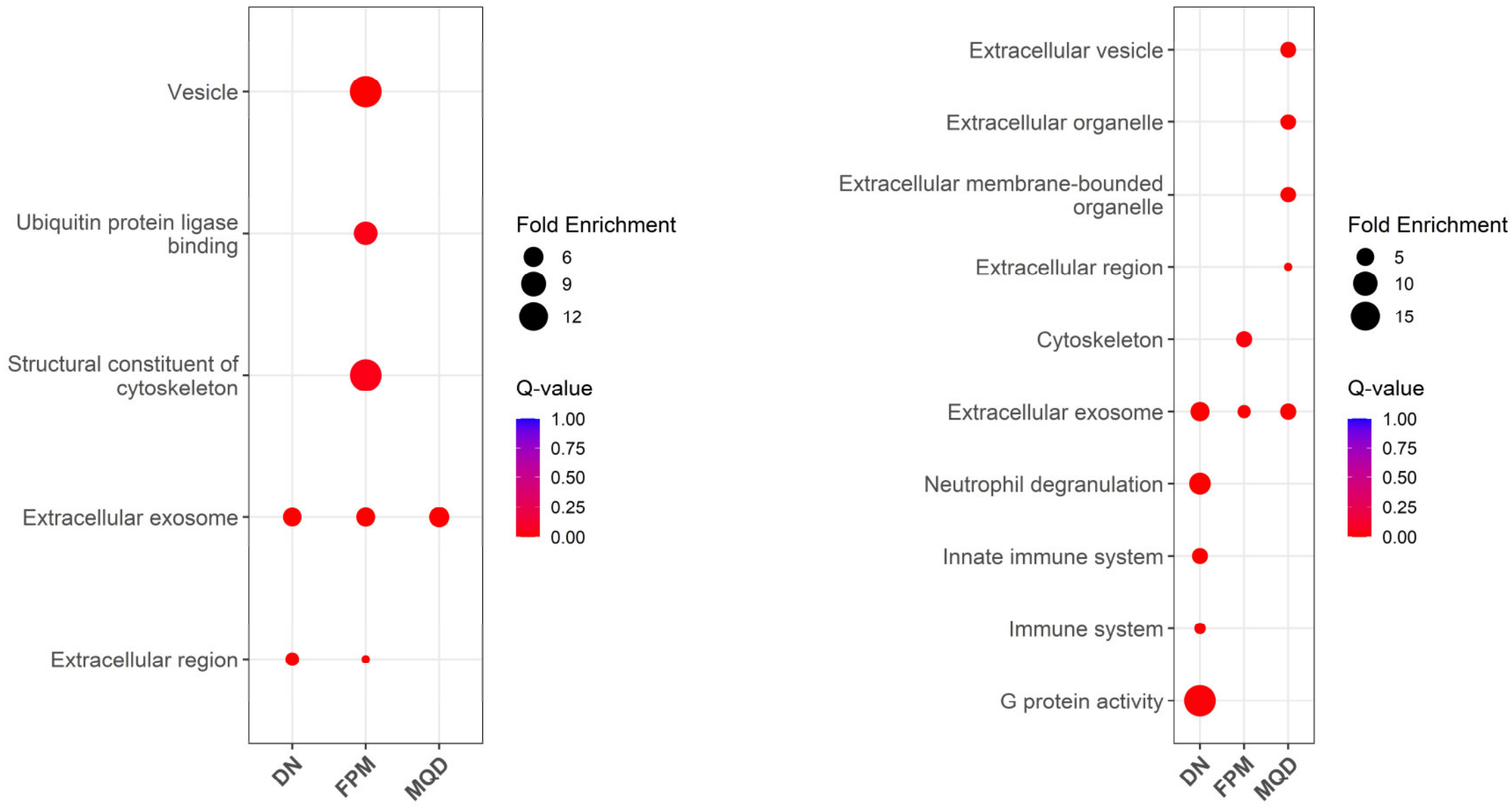

Figure S6. Functional enrichment analysis of tool-specific proteins identified by different DIA tools.

The five most significantly enriched functional terms associated with tool-specific proteins are shown for DIA tools, including DIA-NN (DN), DIA-Umpire (FPD), MSFragger-DIA (FPM), and MaxDIA (MQD) in (A) serum proteome (PXD025462) and (B) plasma proteome (PXD047901).

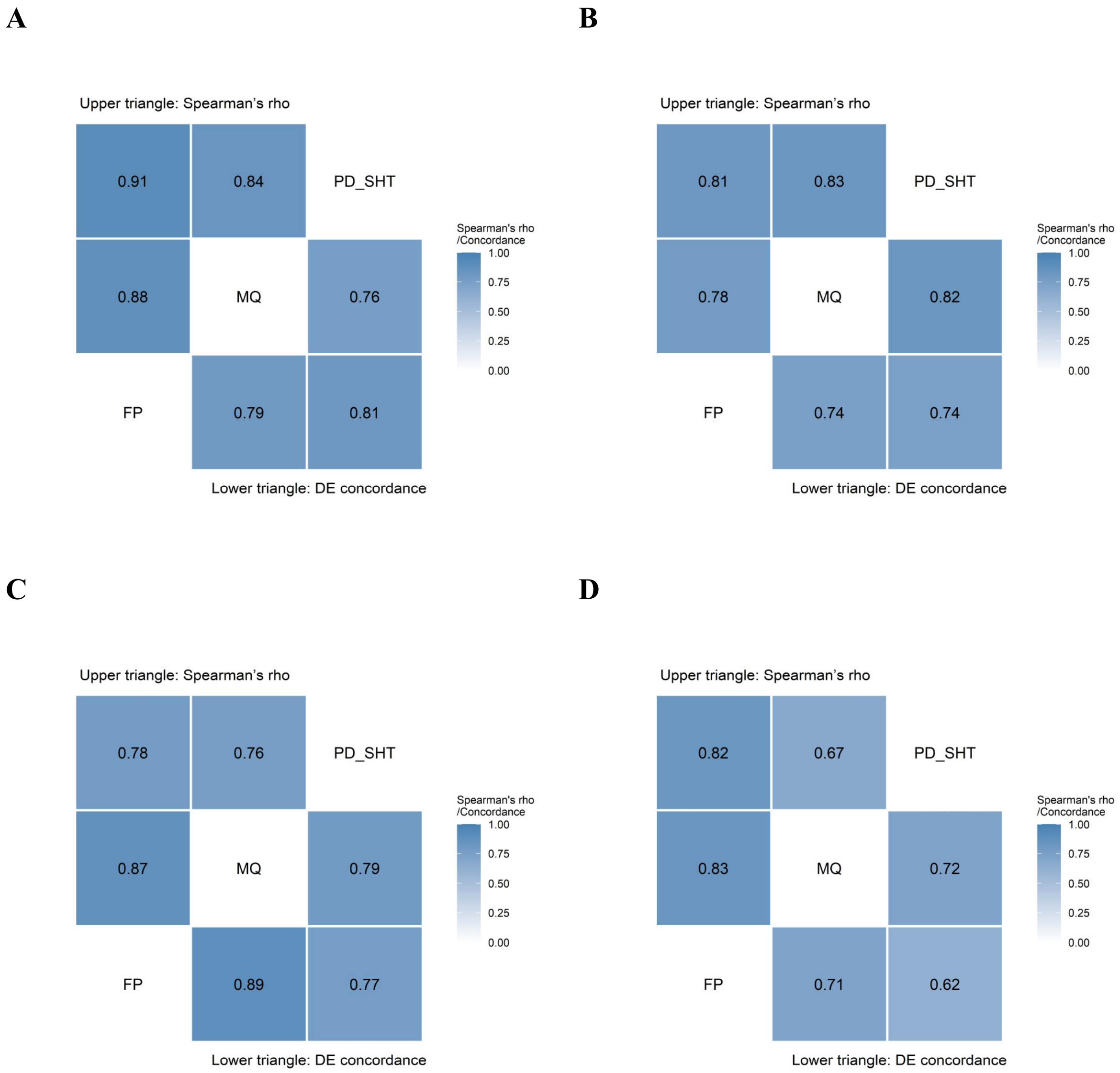

Figure S7. Protein abundance correlations and differential expression directional concordances across label-free DDA tools.

Heatmaps show Spearman rank correlations (ρ) of protein abundances (upper-left triangle) and directional concordances of differential expression results (lower-right triangle) among FragPipe (FP), MaxQuant (MQ), and Proteome Discoverer with Sequest HT (PD_SHT) for (A) cardiac proteome (PXD053721), (B) cardiac proteome (PXD060431), (C) vascular proteome (PXD035563), (D) core vascular matrisome (PXD005130), (E) soluble vascular matrisome (PXD005130), and (F) serum proteome (PXD008278).

E

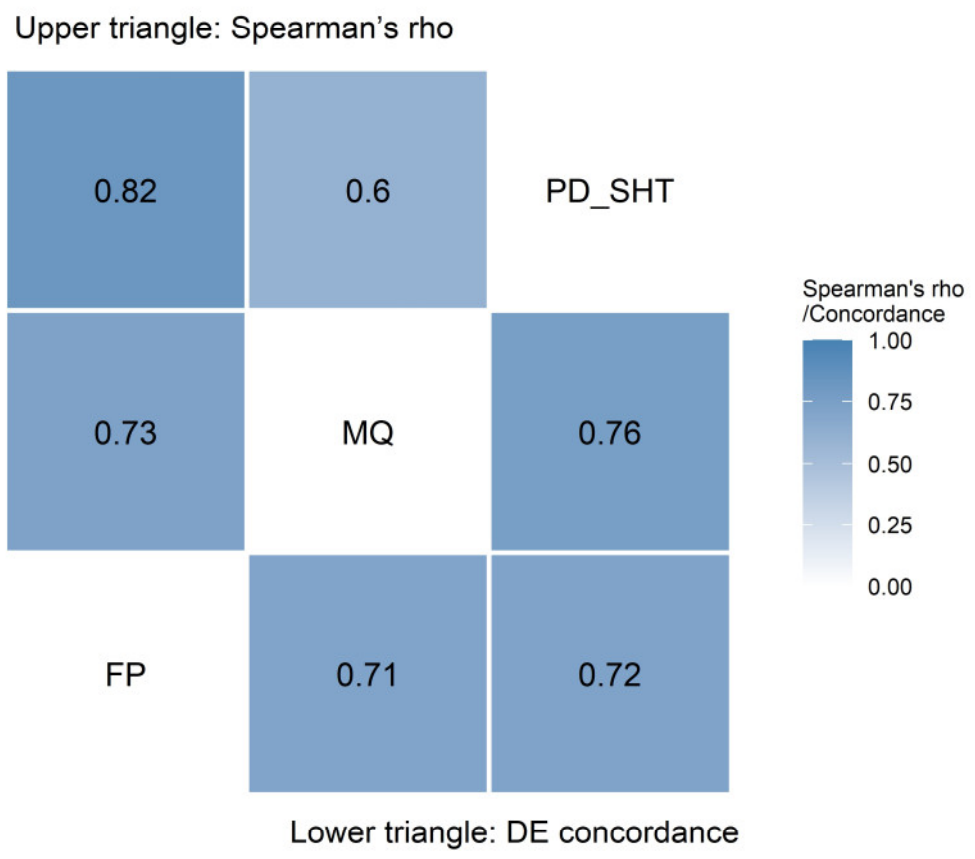

F

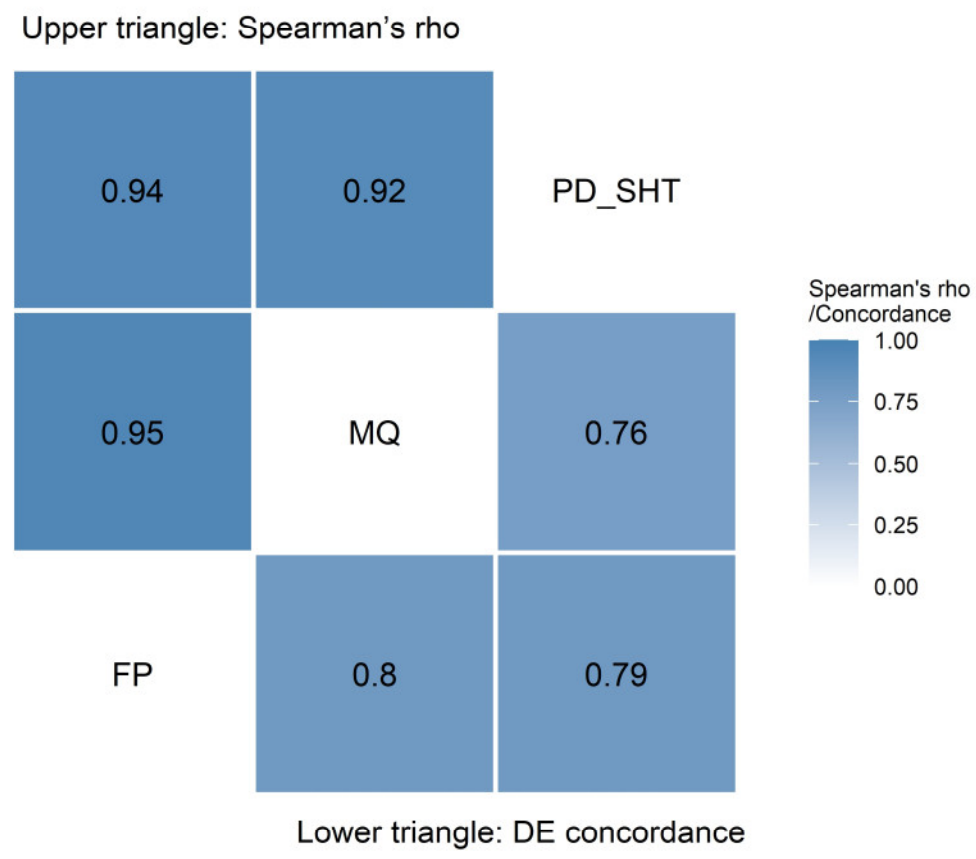

Figure S7 (continued). Protein abundance correlations and differential expression directional concordances across label-free DDA tools.

Heatmaps show Spearman rank correlations (ρ) of protein abundances (upper-left triangle) and directional concordances of differential expression results (lower-right triangle) among FragPipe (FP), MaxQuant (MQ), and Proteome Discoverer with Sequest HT (PD_SHT) for (A) cardiac proteome (PXD053721), (B) cardiac proteome (PXD060431), (C) vascular proteome (PXD035563), (D) core vascular matrisome (PXD005130), (E) soluble vascular matrisome (PXD005130), and (F) serum proteome (PXD008278).

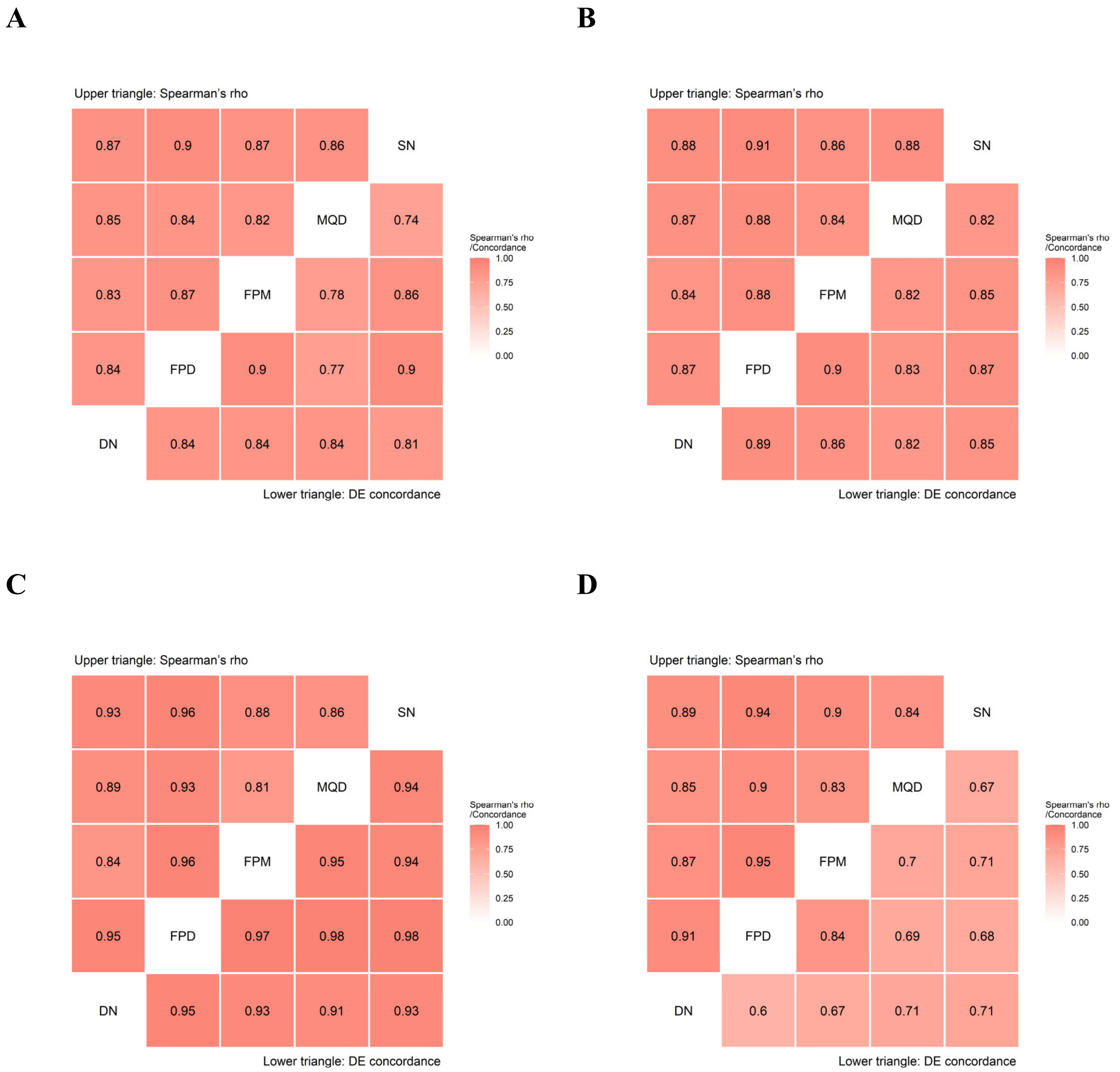

Figure S8. Protein abundance correlations and differential expression directional concordances across DIA tools.
Heatmaps show Spearman rank correlations (ρ) of protein abundances (upper-left triangle) and directional concordances of differential expression results (lower-right triangle) among DIA-NN (DN), DIA-Umpire (FPD), MSFragger-DIA (FPM), MaxDIA (MQD), and Spectronaut (SN) for (A) cardiac proteome (PXD018678), (B) cardiac proteome (PXD050610), (C) serum proteome (PXD025462), (D) plasma proteome (PXD047901), and (E) PQ500-spiked plasma proteome with light-to-heavy peptide ratios (PXD024089).

E

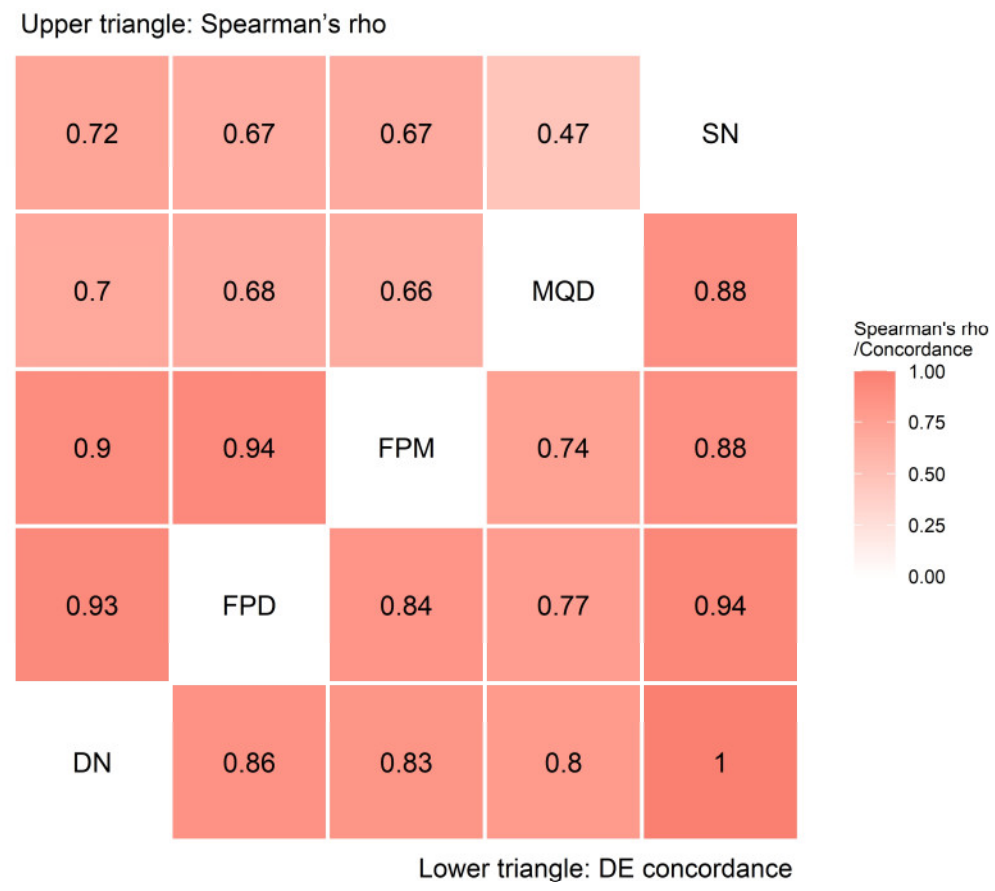

Figure S8 (continued). Protein abundance correlations and differential expression directional concordances across DIA tools.

Heatmaps show Spearman rank correlations (ρ) of protein abundances (upper-left triangle) and directional concordances of differential expression results (lower-right triangle) among DIA-NN (DN), DIA-Umpire (FPD), MSFragger-DIA (FPM), MaxDIA (MQD), and Spectronaut (SN) for (A) cardiac proteome (PXD018678), (B) cardiac proteome (PXD050610), (C) serum proteome (PXD025462), (D) plasma proteome (PXD047901), and (E) PQ500-spiked plasma proteome with light-to-heavy peptide ratios (PXD024089).

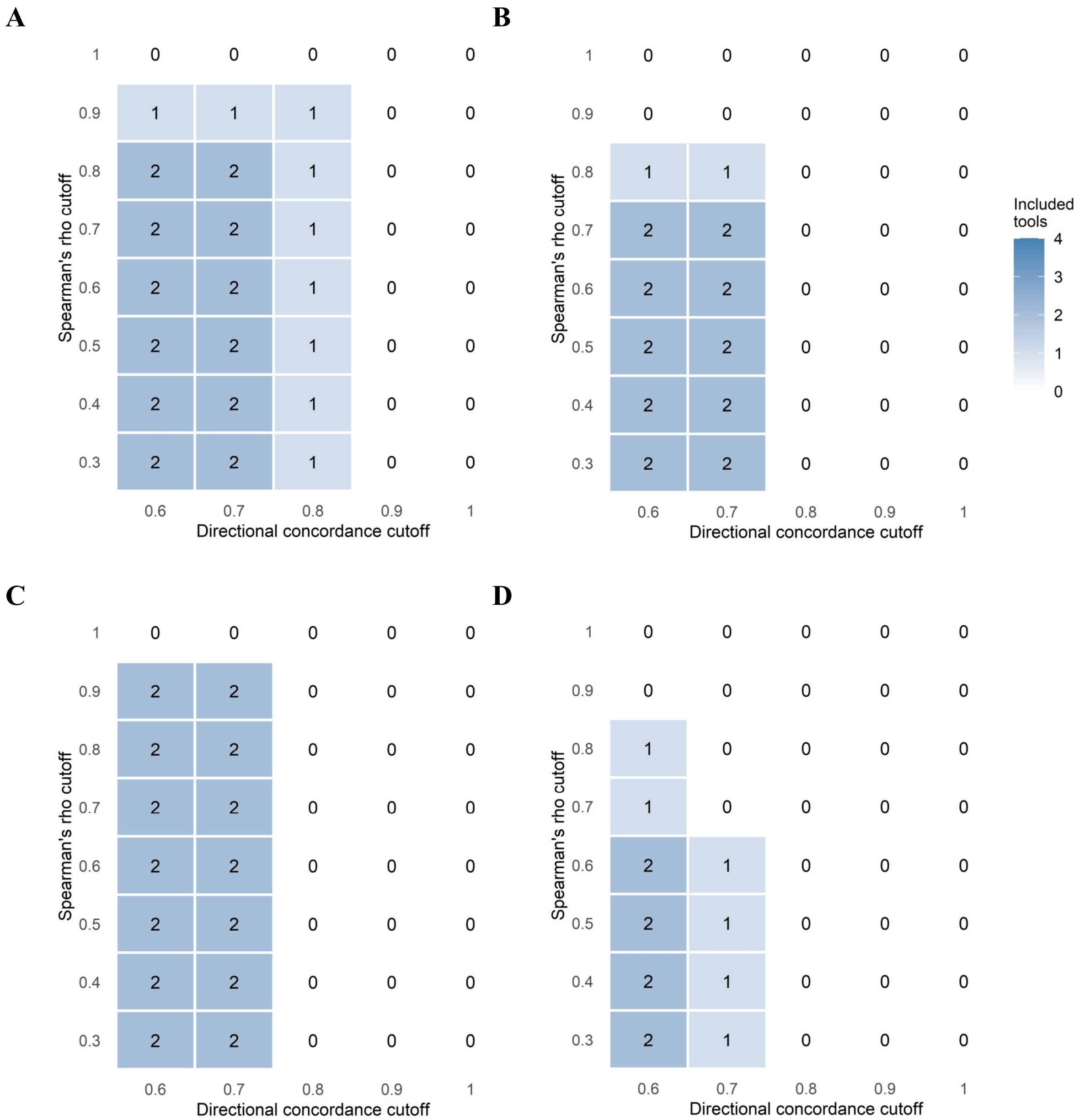

Figure S9. Sensitivity analysis of tool inclusion across agreement thresholds in label-free DDA datasets.

Heatmaps show the number of additional label-free DDA tools meeting the agreement criteria across combinations of Spearman correlation and directional concordance thresholds for (A) cardiac proteome (PXD053721), (B) cardiac proteome (PXD060431), (C) vascular proteome (PXD035563), (D) core vascular matrisome (PXD005130), (E) soluble vascular matrisome (PXD005130), and (F) serum proteome (PXD008278). The primary thresholds used in the study were $\rho > 0.4$ and $\geq 70\%$ directional concordance.

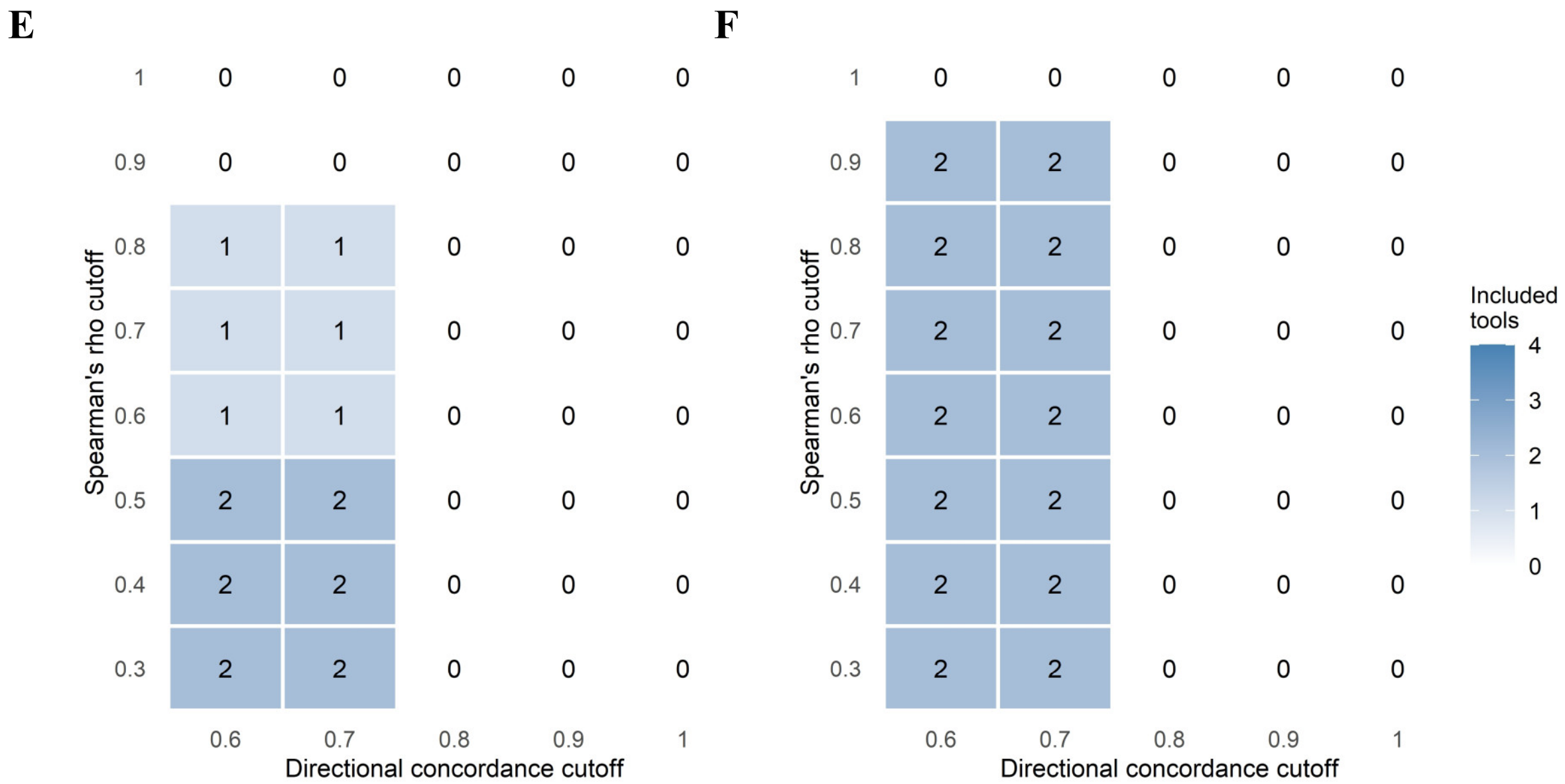

Figure S9 (continued). Sensitivity analysis of tool inclusion across agreement thresholds in label-free DDA datasets.

Heatmaps show the number of additional label-free DDA tools meeting the agreement criteria across combinations of Spearman correlation and directional concordance thresholds for (A) cardiac proteome (PXD053721), (B) cardiac proteome (PXD060431), (C) vascular proteome (PXD035563), (D) core vascular matrisome (PXD005130), (E) soluble vascular matrisome (PXD005130), and (F) serum proteome (PXD008278). The primary thresholds used in the study were $\rho > 0.4$ and ≥70% directional concordance.

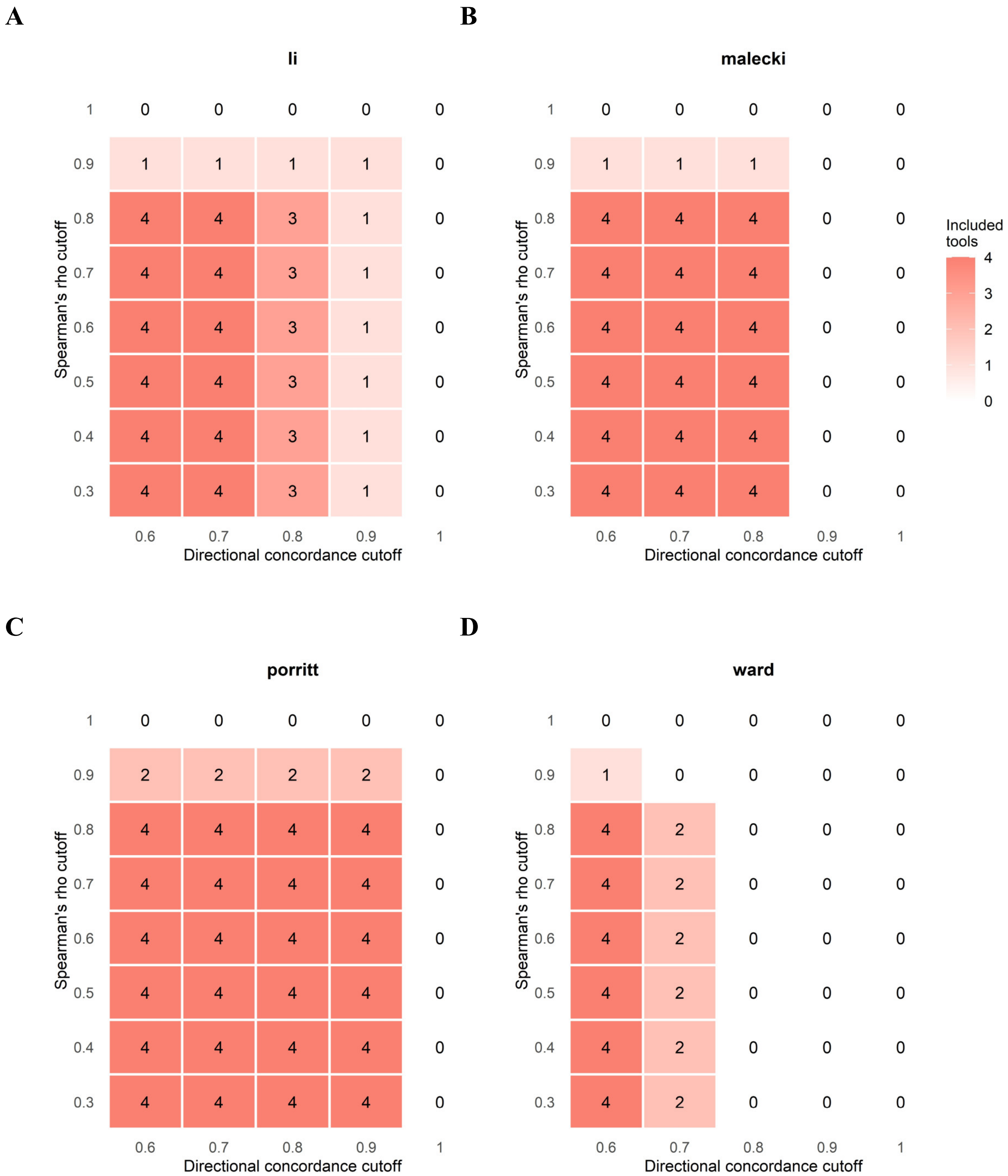

Figure S10. Sensitivity analysis of tool inclusion across agreement thresholds in DIA datasets. Heatmaps show the number of additional DIA tools meeting the agreement criteria across combinations of Spearman correlation and directional concordance thresholds for (A) cardiac proteome (PXD018678), (B) cardiac proteome (PXD050610), (C) serum proteome (PXD025462), (D) plasma proteome (PXD047901), and (E) PQ500-spiked plasma proteome (PXD024089). The primary thresholds used in the study were $\rho > 0.4$ and $\geq$70% directional concordance.

E

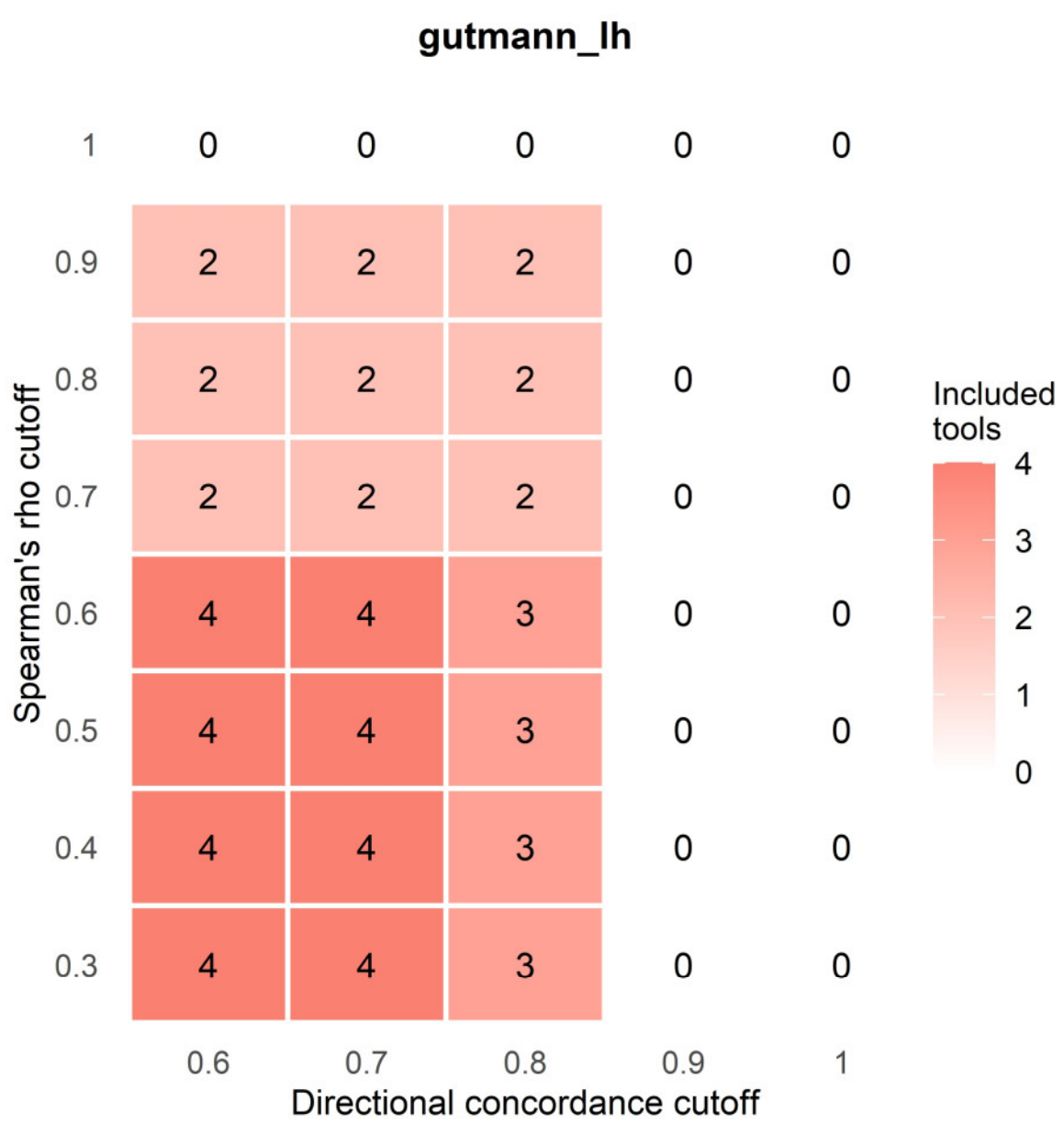

Figure S10 (continued). Sensitivity analysis of tool inclusion across agreement thresholds in DIA datasets.

Heatmaps show the number of additional DIA tools meeting the agreement criteria across combinations of Spearman correlation and directional concordance thresholds for (A) cardiac proteome (PXD018678), (B) cardiac proteome (PXD050610), (C) serum proteome (PXD025462), (D) plasma proteome (PXD047901), and (E) PQ500-spiked plasma proteome (PXD024089). The primary thresholds used in the study were $\rho > 0.4$ and ≥70% directional concordance.

Table S13. Changes in DEA input proteins and significant DEPs following multi-tool integration.

Dataset	Main Tool	DEA input proteins				Significant DEPs			
		Main (n)	Added (n)	Integrated (n)	Increase (%)	Main (n)	Added (n)	Integrated (n)	Increase (%)
**Label-free DDA datasets**									
Cardiac proteome (PXD053721)	FragPipe	3,894	127	4,021	3.3	943	31	974	3.3
Cardiac proteome (PXD060431)	FragPipe	1,748	216	1,964	12.4	4	1	5	25
Vascular proteome (PXD035563)	FragPipe	1,453	73	1,526	5	474	29	503	6.1
Core vascular matrisome (PXD005130)	PD_SHT	1,155	18	1,173	1.6	0	0	0	NA
Soluble vascular matrisome (PXD005130)	PD_SHT	1,512	50	1,562	3.3	10	0	0	0
Serum proteome (PXD008278)	FragPipe	198	34	232	17.2	0	0	0	NA
**DIA datasets**									
Cardiac proteome (PXD018678)	Spectronaut	4,165	364	4,529	8.7	27	5	32	18.5
Cardiac proteome (PXD050610)	Spectronaut	3,652	232	3,884	6.4	19	2	21	10.5
Serum proteome (PXD025462)	Spectronaut	383	90	473	23.5	189	37	226	19.6
Plasma proteome (PXD047901)	DIA-NN	728	63	791	8.7	0	1	1	NA
PQ500-spiked plasma proteome (PXD024089)	Spectronaut	33	102	135	309.1	10	18	28	180

DEA input proteins, quantifiable proteins retained for differential expression analysis; significant DEPs, differentially expressed proteins defined as proteins with q-value < 0.05 and absolute log2 fold-change > 0.3.

A

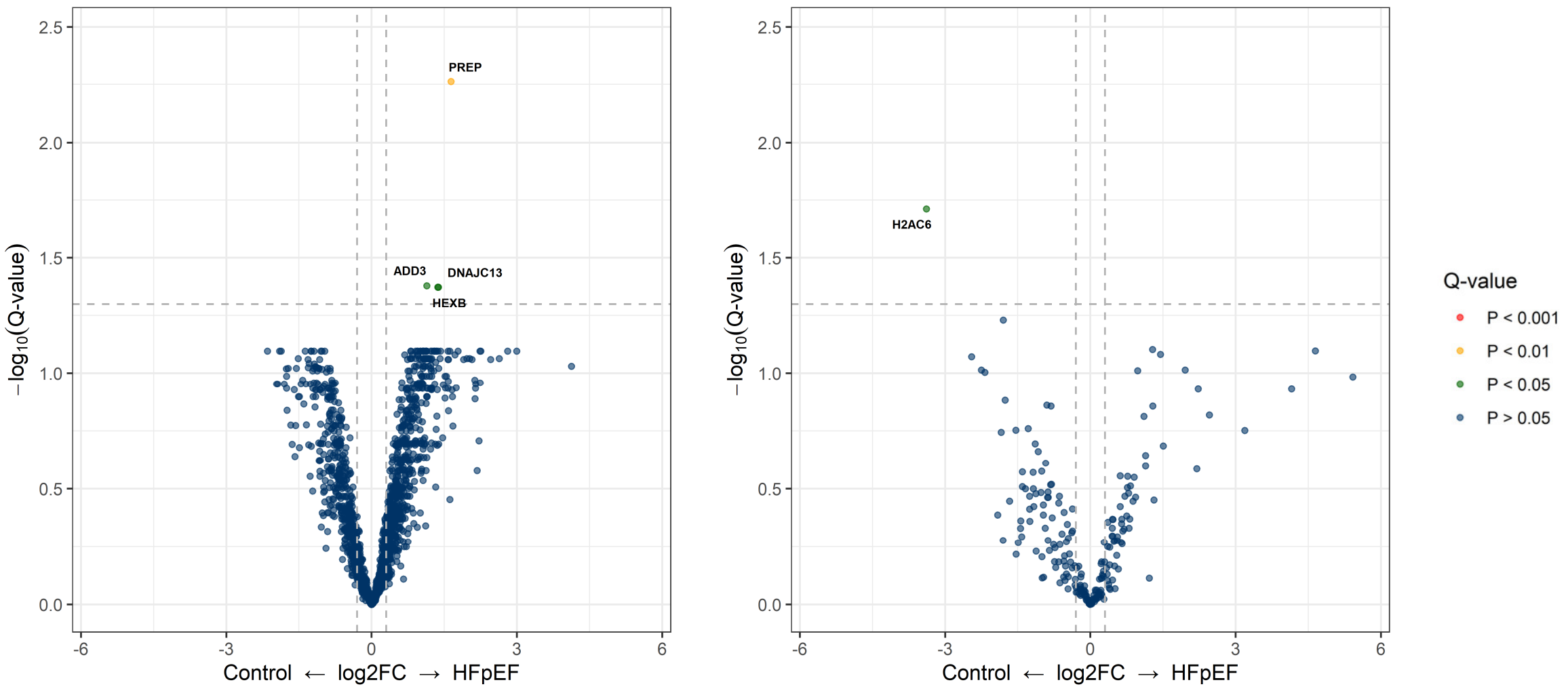

B

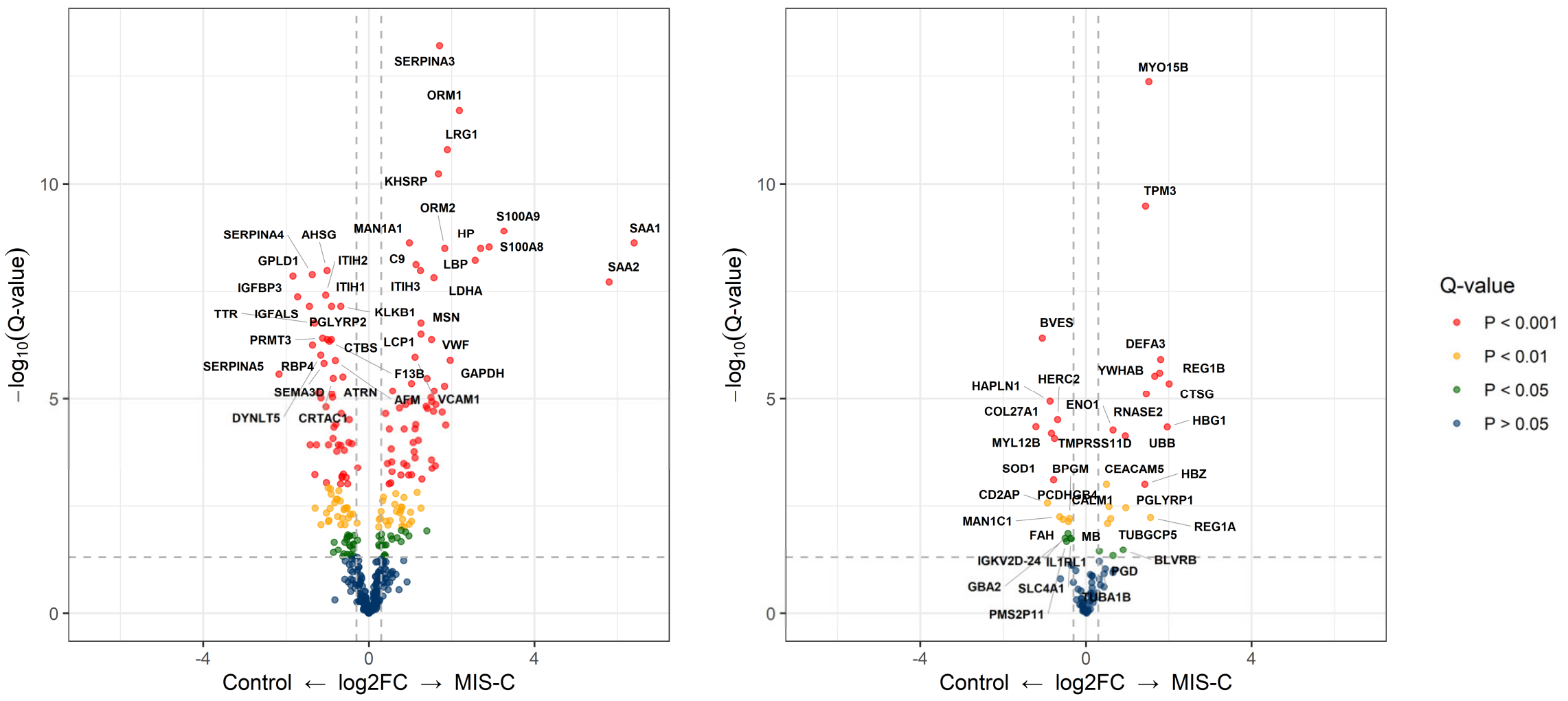

Figure S11. Volcano plots of differentially expressed proteins in label-free DDA and DIA datasets. Plots show log2 fold change versus -log10 Q-value, with the top 40 significant proteins labeled. Left panels show the main differential expression results and right panels show complementary results. Comparisons include (A) 10 heart failure with preserved ejection fraction patients versus 10 non-failing controls (cardiac proteome, PXD060431), (B) 25 MIS-C patients versus 24 healthy controls (serum proteome, PXD025462), (C) 48 COVID-19 ICU patients versus 11 sepsis ICU patients (PQ500-spiked plasma proteome, PXD024089), and (D) 8 severe COVID-19 patients versus 4 respiratory failure controls (plasma proteome, PXD047901).

C

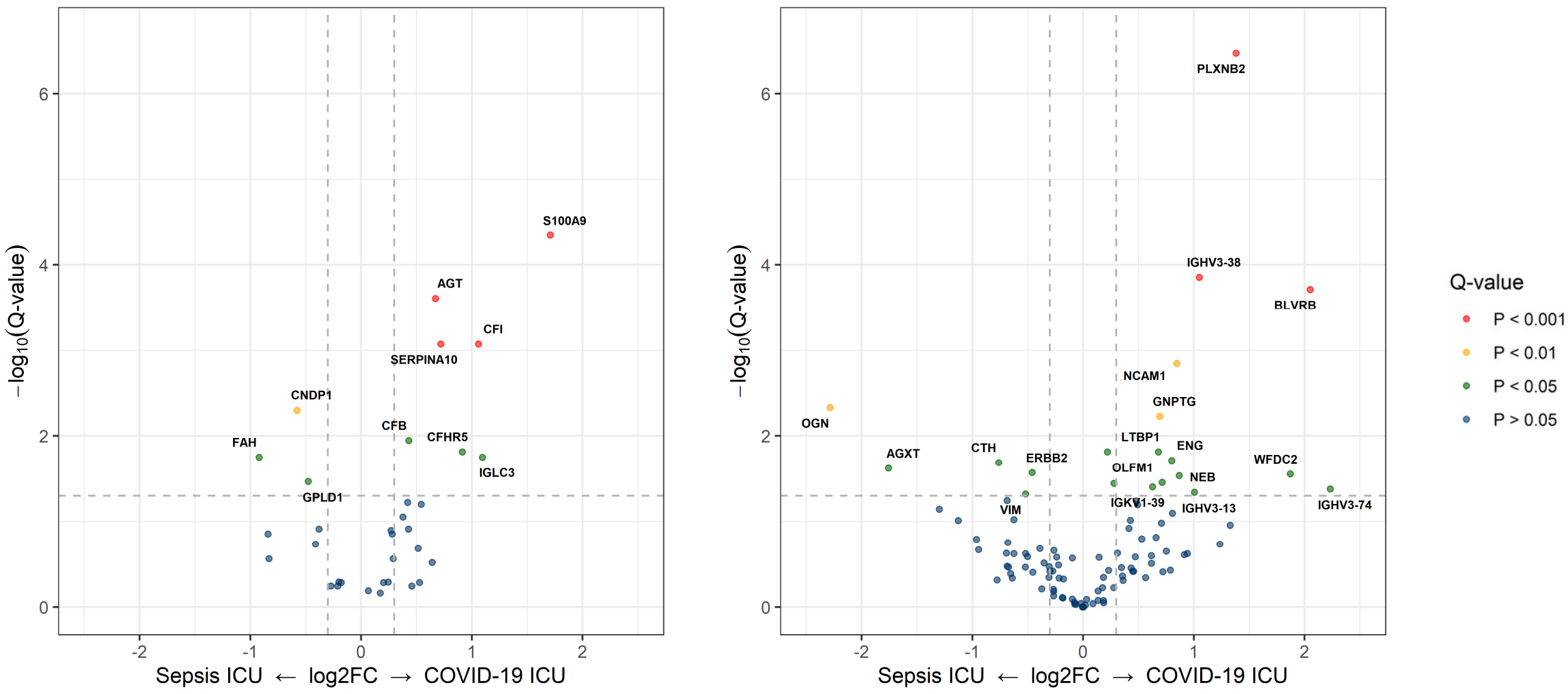

D

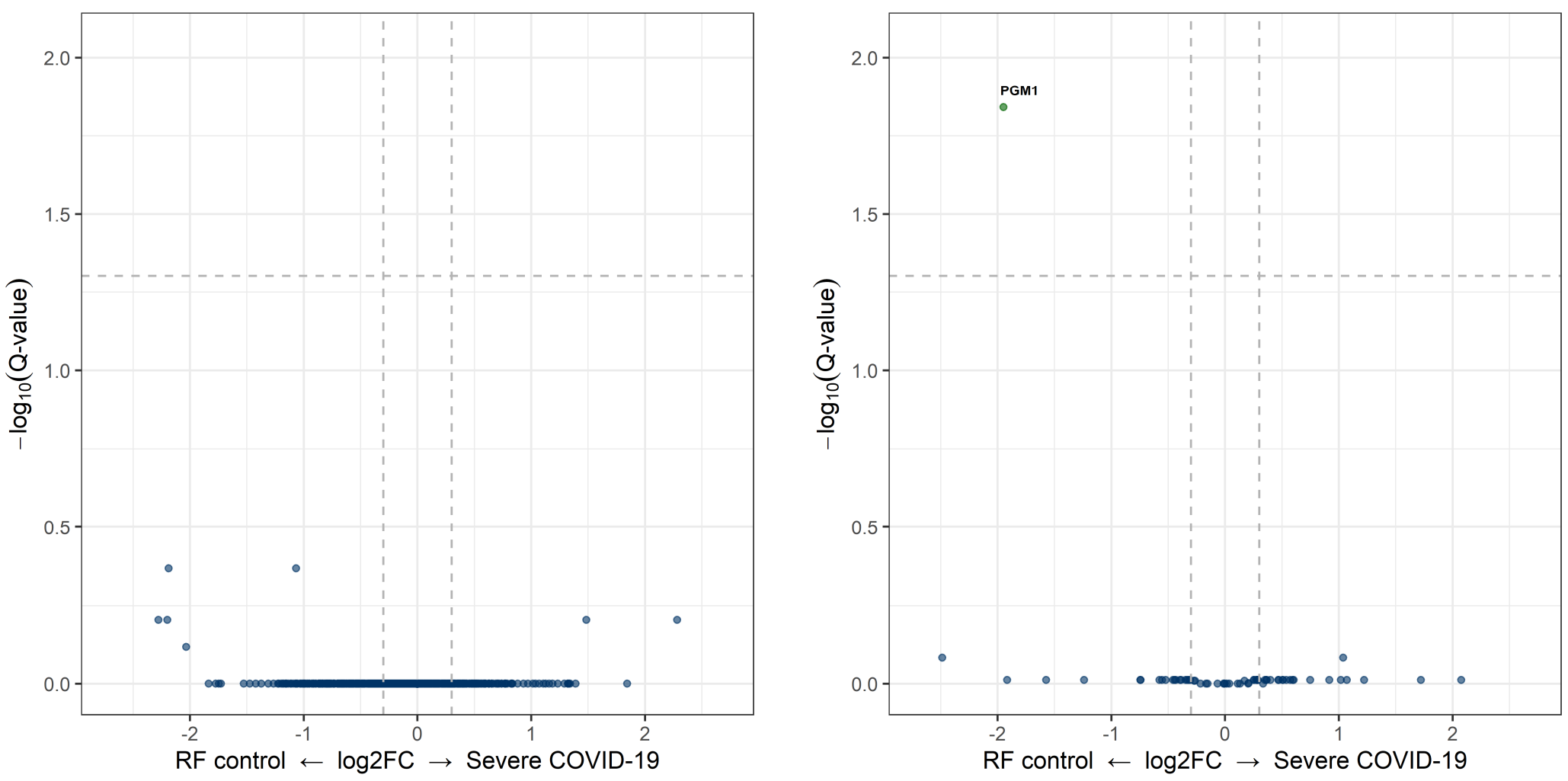

Figure S11 (continued). Volcano plots of differentially expressed proteins in label-free DDA and DIA datasets.

Plots show log2 fold change versus -log10 Q-value, with the top 40 significant proteins labeled. Left panels show the main differential expression results and right panels show complementary results. Comparisons include (A) 10 heart failure with preserved ejection fraction patients versus 10 non-failing controls (cardiac proteome, PXD060431), (B) 25 MIS-C patients versus 24 healthy controls (serum proteome, PXD025462), (C) 48 COVID-19 ICU patients versus 11 sepsis ICU patients (PQ500-spiked plasma proteome, PXD024089), and (D) 8 severe COVID-19 patients versus 4 respiratory failure controls (plasma proteome, PXD047901).

Table S14. Additional significantly dysregulated proteins in the cardiac proteome DDA dataset (PXD053721).

Protein	logFC	AveExpr	t	P.Value	adj.P.Val	B
**HMBS**	0.887511	25.61591	6.386207	1.87E-08	2.69E-06	9.200133
**H2AX**	0.990247	27.70756	6.15032	4.85E-08	5.43E-06	8.283076
**TBCEL**	0.829583	24.7323	5.792903	2.02E-07	1.57E-05	6.911209
**ZNF800**	-1.31103	26.1479	-5.66616	3.34E-07	2.20E-05	6.430757
**STARD9**	0.696003	28.30571	4.8438	7.86E-06	0.000243	3.412806
**LHPP**	0.744815	24.88261	4.189441	8.36E-05	0.001514	1.173226
**NUDT2**	0.725407	23.97551	3.965219	0.000181	0.002747	0.447848
**CROCC**	-0.68229	26.19584	-3.57883	0.000647	0.006553	-0.7432
**PGAP1**	0.726369	24.43767	3.559055	0.00069	0.006944	-0.802
**RPL39P5**	-0.52043	26.33837	-3.37831	0.001219	0.010277	-1.32905
**HEATR5A**	0.665637	23.39977	3.267383	0.001714	0.013425	-1.64297
**SDHAF2**	0.726908	27.37321	3.247684	0.001819	0.014	-1.69792
**IGLC3**	1.068182	27.69341	3.232836	0.001903	0.014495	-1.73919
**SUMO4**	-0.57689	26.42261	-3.21004	0.002038	0.015328	-1.80228
**IGKV1-33**	0.719901	24.69144	3.145364	0.002474	0.017477	-1.97951
**EEF1A1P5**	0.487793	28.59575	3.07681	0.003029	0.020092	-2.16444
**PRXL2B**	0.543381	23.87268	3.024626	0.003527	0.022268	-2.30318
**ORMDL1**	-0.3713	23.96768	-3.00503	0.003733	0.02323	-2.35482
**AKR1C1**	0.634823	26.22113	2.985908	0.003945	0.024088	-2.40495
**TAOK1**	0.346995	24.48912	2.984999	0.003956	0.024088	-2.40733
**RIC1**	0.559208	24.11258	2.965434	0.004184	0.025207	-2.45837
**SH3BGRL2**	-0.3998	23.85742	-2.87145	0.005465	0.030433	-2.69994
**H2AZ2**	-0.61206	28.63124	-2.7934	0.006793	0.036295	-2.89597
**COPS7B**	0.441116	24.29062	2.746149	0.007735	0.03958	-3.01257
**NUP85**	0.473596	23.54279	2.650435	0.010021	0.04761	-3.2439
**IGKV2-40**	1.478787	25.55521	5.926848	1.24E-07	6.17E-06	7.322703
**CYTH3**	0.399842	22.22249	4.600344	1.96E-05	0.000331	2.443171
**IFNGR1**	-0.59393	23.89892	-3.84864	0.00027	0.002433	-0.04626
**DECR2**	-1.55059	25.68349	-3.44267	0.001004	0.007031	-1.27527
**RPS27A**	-0.34179	29.24105	-3.03927	0.003396	0.017689	-2.40112
**ATP5F1E**	-0.64351	25.54954	-2.90497	0.004992	0.023665	-2.75278

Table S15. Additional significantly dysregulated proteins in the cardiac proteome DDA dataset (PXD060431).

Protein	logFC	AveExpr	t	P.Value	adj.P.Val	B
**H2AC6**	-3.38708	29.29914	-5.66579	1.16E-05	0.019408	3.323712

Table S16. Additional significantly dysregulated proteins in the vascular proteome DDA dataset (PXD035563).

Protein	logFC	AveExpr	t	P.Value	adj.P.Val	B
**IGKV2-24**	2.175598	19.01395	5.601145	1.92E-06	0.000166	4.948354
**IGHV3-30-5**	2.334011	19.98959	5.149348	8.03E-06	0.000352	3.594946
**IGLV3-25**	2.184349	19.02106	5.039354	1.14E-05	0.000394	3.267622
**IGHV1-46**	1.908659	19.09341	5.012498	1.24E-05	0.000404	3.187877
**H2AC1**	-1.47548	17.77118	-4.97564	1.39E-05	0.000406	3.078549
**SYT13**	1.745576	18.41493	4.836813	2.14E-05	0.000454	2.668173
**IGKV1-33**	1.938801	18.84524	4.755218	2.76E-05	0.000535	2.428101
**IGKV2D-40**	2.266152	21.12524	4.695157	3.33E-05	0.000571	2.251976
**IGLV3-21**	1.740809	19.82027	4.575908	4.82E-05	0.000707	1.903928
**ACTC1**	-1.30272	29.09954	-4.54708	5.27E-05	0.000754	1.820141
**ARF1**	1.338143	19.38831	4.392671	8.47E-05	0.00102	1.373888
**CD163**	2.230748	18.37728	4.363487	9.26E-05	0.001092	1.29006
**CALCOCO1**	1.313577	20.50757	4.12831	0.000189	0.001912	0.621268
**MYL1**	-1.14927	18.66935	-4.1014	0.000205	0.002004	0.545579
**CAMK2G**	-1.29815	18.95753	-4.09142	0.000211	0.00203	0.517535
**IGKV3-15**	1.40168	20.98108	3.968515	0.000305	0.002527	0.174494
**IGKV3-11**	1.184729	21.36661	3.886145	0.000389	0.002928	-0.05309
**MYL12A**	-0.93748	21.78633	-3.49986	0.001192	0.006461	-1.09191
**ANXA3**	-1.26259	16.61572	-3.47126	0.001292	0.006813	-1.16677
**ITGAX**	1.615669	16.89899	3.190191	0.002824	0.012231	-1.88492
**SPTBN4**	1.41291	18.7488	3.189021	0.002833	0.012231	-1.88784
**HRAS**	-0.58519	18.00222	-3.13156	0.003312	0.013728	-2.03047
**H2AZ2**	-1.3759	19.0428	-3.11289	0.003484	0.014335	-2.07649
**DEFA3**	1.045306	22.8011	2.994028	0.004788	0.018504	-2.36566
**RNPS1**	0.978896	19.12128	2.782062	0.008317	0.028316	-2.8639
**SAR1B**	0.536133	17.49202	2.623746	0.012394	0.038115	-3.22034
**KLHL2**	1.058885	23.90633	2.577997	0.013877	0.041999	-3.32069
**IGLC2**	2.134118	23.74658	4.215412	0.000103	0.001267	1.159799
**H2BC12**	-2.36308	22.69395	-3.81637	0.000371	0.002923	-0.02568

Table S17. Additional significantly dysregulated proteins in the cardiac proteome DIA dataset (PXD018678).

Protein	logFC	AveExpr	t	P.Value	adj.P.Val	B
**VSIG8**	1.214067	23.59067	3.697558	0.00085	0.041485	-0.61642
**NDOR1**	1.363192	22.71236	3.641843	0.000988	0.045236	-0.75348
**PLN**	2.200671	23.76565	5.159596	1.41E-05	0.004668	3.138818
**DDX55**	0.844115	24.8654	4.501928	9.12E-05	0.01477	1.419077
**DNAAF1**	0.879432	26.14502	4.026774	0.000345	0.027944	0.197325

Table S18. Additional significantly dysregulated proteins in the cardiac proteome DIA dataset (PXD050610).

Protein	logFC	AveExpr	t	P.Value	adj.P.Val	B
**ANGPTL2**	1.697767	21.17728	4.959611	3.43E-05	0.00796	2.352961
**IGHV3-30-3**	0.946366	26.62631	3.840104	0.000678	0.046012	-0.40824

Table S19. Additional significantly dysregulated proteins in the serum proteome DIA dataset (PXD025462).

Protein	logFC	AveExpr	t	P.Value	adj.P.Val	B
**YWHAB**	1.659137	20.20125	5.829636	3.97E-07	2.95E-06	6.05064
**CTSG**	2.002743	18.27396	5.682636	6.70E-07	4.44E-06	5.536925
**RNASE2**	1.453294	18.93012	5.490446	1.33E-06	7.71E-06	4.869507
**HBG1**	1.958969	22.60019	4.90526	1.02E-05	4.54E-05	2.875987
**HBZ**	1.416104	19.36368	3.839541	0.000346	0.000999	-0.52295
**PGLYRP1**	0.957103	17.30317	3.363623	0.001479	0.003481	-1.89875
**REG1A**	1.558202	18.74108	3.159923	0.002673	0.005819	-2.45297
**PMS2P11**	-0.47243	24.43674	-2.64725	0.010813	0.020595	-3.7408
**BLVRB**	0.892727	18.66377	2.438884	0.01831	0.032917	-4.21562
**DEFA3**	1.799141	22.07865	6.115724	1.46E-07	1.21E-06	6.58228
**TUBGCP5**	0.594596	22.55103	3.236642	0.002151	0.006198	-2.77064
**MYO15B**	1.51521	20.50797	11.15433	3.29E-15	4.20E-13	24.42979
**TPM3**	1.434044	21.25939	8.974251	5.09E-12	3.25E-10	17.19637
**BVES**	-1.05838	19.5537	-6.5459	3.00E-08	3.90E-07	8.641701
**REG1B**	1.777902	18.54654	5.946433	2.60E-07	2.49E-06	6.523857
**HAPLN1**	-0.87172	20.11888	-5.42771	1.65E-06	1.15E-05	4.718553
**HERC2**	-0.68571	20.86119	-5.11343	4.95E-06	3.06E-05	3.644562
**COL27A1**	-1.21231	18.53681	-4.98774	7.66E-06	4.45E-05	3.220421
**ENO1**	0.649616	19.90267	4.92017	9.67E-06	5.37E-05	2.993854
**MYL12B**	-0.83572	23.11725	-4.86797	1.16E-05	6.33E-05	2.819532
**UBB**	0.946484	18.79542	4.807687	1.42E-05	7.30E-05	2.61905
**SOD1**	-0.78644	21.65172	-4.01329	0.000199	0.000787	0.075931
**CEACAM5**	0.490006	18.17047	3.910845	0.000276	0.001007	-0.23609
**CD2AP**	-0.934	17.61915	-3.53074	0.000897	0.002705	-1.35458
**CALM1**	0.555083	18.3329	3.444161	0.001163	0.003276	-1.59997
**MAN1C1**	-0.63629	24.24346	-3.21746	0.002264	0.005631	-2.22432
**BPGM**	-0.39562	20.13342	-3.17928	0.002527	0.00614	-2.32677
**FAH**	-0.55956	19.26352	-3.15714	0.002692	0.006484	-2.3858
**PCDHGB4**	-0.42327	20.05424	-3.11242	0.003057	0.007273	-2.50421
**MB**	0.519314	18.14248	3.069867	0.003448	0.008002	-2.61581
**IGKV2D-24**	-0.43731	18.09666	-2.86684	0.00604	0.013608	-3.13358
**GBA2**	-0.5035	20.67173	-2.74324	0.008408	0.017566	-3.43634

Table S18 (continued).

Protein	logFC	AveExpr	t	P.Value	adj.P.Val	B
**SLC4A1**	-0.37313	23.26081	-2.74181	0.008439	0.017566	-3.43977
**IL1RL1**	-0.36048	19.38831	-2.72973	0.008713	0.01794	-3.46882
**TUBA1B**	0.320827	18.81665	2.437096	0.018383	0.035381	-4.14281
**PGD**	0.649164	18.99373	2.323829	0.024213	0.045018	-4.38762
**TMPRSS11D**	-0.76471	21.99199	-4.65412	2.47E-05	8.46E-05	2.071497

Table S20. Additional significantly dysregulated proteins in the PQ500-spiked plasma proteome DIA dataset (PXD024089).

Protein	logFC	AveExpr	t	P.Value	adj.P.Val	B
**PLXNB2**	1.379605	-0.66234	7.095972	1.70E-09	3.31E-07	11.53446
**IGHV3-38**	1.049264	2.202144	5.133554	3.23E-06	0.00014	4.288842
**BLVRB**	2.050447	-2.73624	4.904591	7.46E-06	0.000194	3.48864
**NCAM1**	0.846318	-2.04382	4.140566	0.00011	0.001427	0.940044
**GNPTG**	0.691098	-4.04551	3.617431	0.000611	0.005954	-0.66808
**ENG**	0.79997	-3.08085	3.058323	0.003321	0.019331	-2.22976
**AGXT**	-1.75674	-3.24827	-2.94325	0.00461	0.023919	-2.52812
**WFDC2**	1.870869	-8.0459	2.857323	0.00586	0.028006	-2.74535
**OLFM1**	0.712685	-2.1726	2.757208	0.007709	0.03496	-2.99229
**IGKV1-39**	0.626421	3.715134	2.685204	0.009355	0.03955	-3.16569
**IGHV3-74**	2.230897	2.462816	2.652788	0.010196	0.041857	-3.24258
**IGHV3-13**	1.004488	6.915486	2.596798	0.011813	0.045613	-3.37367
**VIM**	-0.51963	-4.95799	-2.57174	0.012609	0.047661	-3.43161
**OGN**	-2.28637	-1.08555	-3.6751	0.000509	0.004696	-0.51719
**LTBP1**	0.679939	-5.24827	3.130306	0.002696	0.015405	-2.06053
**CTH**	-0.76395	-3.01678	-2.97533	0.004212	0.02049	-2.46783
**ERBB2**	-0.46052	-5.15105	-2.85508	0.005899	0.02688	-2.77332
**NEB**	0.867598	1.276345	2.803327	0.006801	0.029175	-2.90188

Table S21. Additional significantly dysregulated proteins in the plasma proteome DIA dataset (PXD047901).

Protein	logFC	AveExpr	t	P.Value	adj.P.Val	B
**PGM1**	-1.9496	16.82004	-5.76561	2.33E-05	0.014403	0.249817

References

(1) Beyhoff, N.; Braun, V. M.; Kirchner, M.; Finnigan, L. E. M.; Knosalla, C.; Baczkó, I.; Potapov, E.; Kintscher, U.; Grune, T.; Kuehne, T.; et al. Computational modelling of myocardial metabolism in patients with advanced heart failure. In *Eur. J. Heart Fail.*, 20250715 ed.; 2025.

(2) Jani, V. P.; Yoo, E. J.; Binek, A.; Guo, A.; Kim, J. S.; Aguilan, J.; Keykhaei, M.; Jenkin, S. R.; Sidoli, S.; Sharma, K.; et al. Myocardial proteome in human heart failure with preserved ejection fraction. *J Am Heart Assoc* **2025**, *14* (6), e038945. DOI: 10.1161/jaha.124.038945 From NLM.

(3) Blaser, M. C.; Buffolo, F.; Halu, A.; Turner, M. E.; Schlotter, F.; Higashi, H.; Pantano, L.; Clift, C. L.; Saddic, L. A.; Atkins, S. K.; et al. Multiomics of tissue extracellular vesicles identifies unique modulators of atherosclerosis and calcific aortic valve stenosis. *Circulation* **2023**, *148* (8), 661–678. DOI: 10.1161/CIRCULATIONAHA.122.063402 (accessed 2025/08/04).

(4) Langley, S. R.; Willeit, K.; Didangelos, A.; Matic, L. P.; Skroblin, P.; Barallobre-Barreiro, J.; Lengquist, M.; Rungger, G.; Kapustin, A.; Kedenko, L.; et al. Extracellular matrix proteomics identifies molecular signature of symptomatic carotid plaques. *J Clin Invest* **2017**, *127* (4), 1546–1560. DOI: 10.1172/jci86924 From NLM.

(5) Bhosale, S. D.; Moulder, R.; Venäläinen, M. S.; Koskinen, J. S.; Pitkänen, N.; Juonala, M. T.; Kähönen, M. A. P.; Lehtimäki, T. J.; Viikari, J. S. A.; Elo, L. L.; et al. Serum proteomic profiling to identify biomarkers of premature carotid atherosclerosis. *Sci Rep* **2018**, *8* (1), 9209. DOI: 10.1038/s41598-018-27265-9.

(6) Li, M.; Parker, B. L.; Pearson, E.; Hunter, B.; Cao, J.; Koay, Y. C.; Guneratne, O.; James, D. E.; Yang, J.; Lal, S.; et al. Core functional nodes and sex-specific pathways in human ischaemic and dilated cardiomyopathy. *Nat Commun* **2020**, *11* (1), 2843. DOI: 10.1038/s41467-020-16584-z From NLM.

(7) Malecki, C.; Guglielmi, G.; Hunter, B.; Harney, D.; Koay, Y. C.; Don, A. S.; Han, O.; Khor, J.; Nguyen, L.; Pan, M.; et al. The human cardiac "Age-OME": age-specific changes in myocardial molecular expression. *Aging Cell* **2025**, *24* (11), e70219. DOI: 10.1111/acel.70219 From NLM.

(8) Porritt, R. A.; Binek, A.; Paschold, L.; Rivas, M. N.; McArdle, A.; Yonker, L. M.; Alter, G.; Chandnani, H. K.; Lopez, M.; Fasano, A.; et al. The autoimmune signature of hyperinflammatory multisystem inflammatory syndrome in children. *J Clin Invest* **2021**, *131* (20). DOI: 10.1172/jci151520 From NLM.

(9) Ward, B.; Pyr Dit Ruys, S.; Balligand, J. L.; Belkhir, L.; Cani, P. D.; Collet, J. F.; De Greef, J.; Dewulf, J. P.; Gatto, L.; Haufroid, V.; et al. Deep plasma proteomics with data-independent acquisition: clinical study protocol optimization with a COVID-19 cohort. *J Proteome Res* **2024**, *23* (9), 3806–3822. DOI: 10.1021/acs.jproteome.4c00104 From NLM.

(10) Gutmann, C.; Takov, K.; Burnap, S. A.; Singh, B.; Ali, H.; Theofilatos, K.; Reed, E.; Hasman, M.; Nabeebaccus, A.; Fish, M.; et al. SARS-CoV-2 RNAemia and proteomic trajectories inform prognostication in COVID-19 patients admitted to intensive care. *Nat Commun* **2021**, *12* (1), 3406. DOI: 10.1038/s41467-021-23494-1 From NLM.
